# Gene–gene interaction of *AhR*with and within the *Wnt*cascade affects susceptibility to lung cancer

**DOI:** 10.1186/s40001-022-00638-7

**Published:** 2022-01-31

**Authors:** Albert Rosenberger, Nils Muttray, Rayjean J. Hung, David C. Christiani, Neil E. Caporaso, Geoffrey Liu, Stig E. Bojesen, Loic Le Marchand, Demetrios Albanes, Melinda C. Aldrich, Adonina Tardon, Guillermo Fernández-Tardón, Gad Rennert, John K. Field, Michael P. A. Davies, Triantafillos Liloglou, Lambertus A. Kiemeney, Philip Lazarus, Bernadette Wendel, Aage Haugen, Shanbeh Zienolddiny, Stephen Lam, Matthew B. Schabath, Angeline S. Andrew, Eric J. Duell, Susanne M. Arnold, Gary E. Goodman, Chu Chen, Jennifer A. Doherty, Fiona Taylor, Angela Cox, Penella J. Woll, Angela Risch, Thomas R. Muley, Mikael Johansson, Paul Brennan, Maria Teresa Landi, Sanjay S. Shete, Christopher I. Amos, Heike Bickeböller, The INTEGRAL-ILCCO Consortium

**Affiliations:** 1 Department of Genetic Epidemiology, University Medical Center, Georg-August-University Göttingen, Göttingen, Germany; 2 Lunenfeld-Tanenbaum Research Institute, Sinai Health System, University of Toronto, Toronto, ON Canada; 3 Dalla Lana School of Public Health, University of Toronto, Toronto, Canada; 4 Department of Environmental Health, Harvard T.H. Chan School of Public Health and Massachusetts General Hospital/Harvard Medical School, Boston, MA USA; 5 Division of Cancer Epidemiology and Genetics, National Cancer Institute, US National Institutes of Health, Bethesda, MD USA; 6 Medical Oncology and Medical Biophysics, Princess Margaret Cancer Centre, Toronto, ON Canada; 7 Medicine and Epidemiology, Dalla Lana School of Public Health, University of Toronto, Toronto, ON Canada; 8 Department of Clinical Biochemistry, Herlev and Gentofte Hospital, Copenhagen University Hospital, Copenhagen, Denmark; 9 Faculty of Health and Medical Sciences, University of Copenhagen, Copenhagen, Denmark; 10 Copenhagen General Population Study, Herlev and Gentofte Hospital, Copenhagen, Denmark; 11 Epidemiology Program, University of Hawaii Cancer Center, Honolulu, HI USA; 12 Department of Thoracic Surgery, Division of Epidemiology, Vanderbilt University Medical Center, Nashville, TN USA; 13 Faculty of Medicine, University of Oviedo, ISPA and CIBERESP, Oviedo, Spain; 14 Clalit National Cancer Control Center at Carmel Medical Center and Technion Faculty of Medicine, Haifa, Israel; 15 Department of Molecular and Clinical Cancer Medicine, Roy Castle Lung Cancer Research Programme, The University of Liverpool, Liverpool, UK; 16 Departments of Health Evidence and Urology, Radboud University Medical Center, Nijmegen, The Netherlands; 17 Department of Pharmaceutical Sciences, College of Pharmacy, Washington State University, Spokane, WA USA; 18 National Institute of Occupational Health, Oslo, Norway; 19 British Columbia Cancer Agency, Vancouver, BC Canada; 20 Department of Cancer Epidemiology, H. Lee Moffitt Cancer Center and Research Institute, Tampa, FL USA; 21 Department of Epidemiology, Geisel School of Medicine, Hanover, NH USA; 22 Unit of Biomarkers and Susceptibility, Oncology Data Analytics Program, Catalan Institute of Oncology (ICO), Bellvitge Biomedical Research Institute (IDIBELL), Barcelona, Spain; 23 Markey Cancer Center, University of Kentucky, Lexington, KY USA; 24 Swedish Medical Group, Seattle, WA USA; 25 Program in Epidemiology, Fred Hutchinson Cancer Research Center, Seattle, WA USA; 26 Department of Population Health Sciences, Huntsman Cancer Institute, University of Utah, Salt Lake City, UT USA; 27 Department of Oncology and Metabolism, University of Sheffield, Sheffield, UK; 28 University of Salzburg and Cancer Cluster Salzburg, Salzburg, Austria; 29 Member of the German Center for Lung Research (DZL), Translational Lung Research Center (TLRC) Heidelberg, Heidelberg, Germany; 30 Translational Research Unit, Thoraxklinik, University Hospital Heidelberg, Heidelberg, Germany; 31 Department of Radiation Sciences, Umeå University, Umeå, Sweden; 32 International Agency for Research on Cancer, World Health Organization, Lyon, France; 33 Department of Biostatistics, Division of Basic Sciences, The University of Texas MD Anderson Cancer Center, Houston, TX USA; 34 Dan L Duncan Comprehensive Cancer Center, Baylor College of Medicine, Houston, TX USA; 35 Institut Für Genetische Epidemiologie, Universitätsmedizin Göttingen, Humboldtallee 32, 37073 Göttingen, Germany

**Keywords:** Susceptibility, Association, Gene–gene integration, Prediction, Polygenic risk score, Decision trees, Never smoker, Small cell lung cancer

## Abstract

**Background:**

Aberrant *Wnt* signalling, regulating cell development and stemness, influences the development of many cancer types. The Aryl hydrocarbon receptor (*AhR*) mediates tumorigenesis of environmental pollutants. Complex interaction patterns of genes assigned to *AhR*/*Wnt*-signalling were recently associated with lung cancer susceptibility.

**Aim:**

To assess the association and predictive ability of *AhR*/*Wnt*-genes with lung cancer in cases and controls of European descent.

**Methods:**

Odds ratios (OR) were estimated for genomic variants assigned to the Wnt agonist and the antagonistic genes *DKK2*, *DKK3*, *DKK4*, *FRZB*, *SFRP4* and *Axin2*. Logistic regression models with variable selection were trained, validated and tested to predict lung cancer, at which other previously identified SNPs that have been robustly associated with lung cancer risk could also enter the model. Furthermore, decision trees were created to investigate variant × variant interaction. All analyses were performed for overall lung cancer and for subgroups.

**Results:**

No genome-wide significant association of *AhR*/*Wnt*-genes with overall lung cancer was observed, but within the subgroups of ever smokers (e.g., maker rs2722278 *SFRP4*; OR  = 1.20; 95% CI 1.13–1.27; *p*  = 5.6 × 10^–10^) and never smokers (e.g., maker rs1133683 *Axin2*; OR  = 1.27; 95% CI 1.19–1.35; *p*  = 1.0 × 10^–12^). Although predictability is poor, *AhR*/*Wnt-variants* are unexpectedly overrepresented in optimized prediction scores for overall lung cancer and for small cell lung cancer. Remarkably, the score for never-smokers contained solely two *AhR*/*Wnt-variants*. The optimal decision tree for never smokers consists of 7 *AhR*/*Wnt-variants* and only two lung cancer variants.

**Conclusions:**

The role of variants belonging to *Wnt*/*AhR-*pathways in lung cancer susceptibility may be underrated in main-effects association analysis. Complex interaction patterns in individuals of European descent have moderate predictive capacity for lung cancer or subgroups thereof, especially in never smokers.

**Supplementary Information:**

The online version contains supplementary material available at 10.1186/s40001-022-00638-7.

## Background

Lung cancer (LC) is the most common cancer worldwide since 1985. It is the leading cause of cancer related death around the world [1]. It was estimated for 2020, that globally 2.2 million new LC-cases were diagnosed, which are 11.4% of all new cancer cases. In the same year 1.8 million LC-cases died, which are 18% of all cancer related deaths [2]. The lifetime risk of developing a clinical manifest lung cancer (from birth to age 74) is higher in men (3.78%) than in women (1.77%).

The *Wnt* signalling pathway is a multi-regulator of, e.g., cell proliferation, differentiation, genetic stability, and much more. It is crucial in the development of embryos and in the dynamic balance of adult tissues, so also that of the lung. With respect to LC, changes of the *Wnt* signalling pathway have been observed for *Wnt* ligands, frizzled, TCF/LEF (T cell factor/lymphoid enhancer factor)-dependent transcription, and *Wnt* inhibitor silencing [3].

Genome-wide association studies (GWAS) have identified dozens of susceptibility loci throughout the genome that are associated with the susceptibility to lung cancer or one of its histological subtypes [4–11]. Genes related to *Wnt* signalling, one of the key pathway regulating cell development and stemness, were not detected as being associated to LC susceptibility in individuals of European descent so far, unlike *TERT* (5p15.33) that was one of the first for which a robust association was observed [12]. Aberrant *Wnt* signalling is often observed in expression profiles of many cancers, but to date no association of *Wnt*/*Ahr* genes with susceptibility to cancer of any type has been observed [13–15]. Administration of RNAi against *Wnt* was shown to reduce tumour burden in lung adenocarcinoma (adenoLC) [16]. In non-small cell lung cancer (NSCLC), overexpressed *miR-582-3p* maintains stemness features by negatively targeting the regulators of *Wnt* signalling *Axin2*, *DKK3* and *SRP1* for degradation, thereby increasing β-catenin mediated *Wnt* activity [17]. *TERT* expression was found to be directly enhanced by binding of β-catenin to its promoter region and thereby links telomerase activity to *Wnt* signalling [13]. This is as much important, as *TERT* is one of the first and most robust susceptibility genes for LC identified by GWAS [18, 19]. The tight regulatory machinery of the *Wnt* pathway has several major antagonists, such as Secreted Frizzled related protein (*sFRP*), Dickopff 5 (*DKK*) protein and *Axin2* protein [20]. Evidence also exists for a crosstalk between *AhR* and *Wnt* signalling [21].

Aryl hydrocarbon receptor (7p21.1; *AhR*) is a ligand induced transcription factor, which is translocated into the nucleus. It is known to mediate the toxicity and tumorigenesis of a variety of environmental pollutants, including for NSCLC. *AhR* upregulates the enzyme CYP1A1 when cells are exposed to carcinogenic metabolites, such as some polycyclic aromatic hydrocarbons (PAHs) found in cigarette smoke. The CYP1A1 coding gene is discussed as a susceptibility gene for LC. *AhR* is a major determinant in the process of smoking driven LC [22–24]. The complexity of both the *AhR* signalling pathway and the *Wnt* signalling cascade is reflected by interaction effects of genomic variants within genes, which control their function [25]. Recently, the association of the *Wnt-*genes *DKK4* (8p11.21), *DKK3* (11p15.3), *DKK2* (4q25), *FRZB* (2q32.1, also known as *sFRP*3), *SFRP4* (7p14.1), *Axin2* (17q24.1) and a potential interaction with *AhR* was investigated with respect to the susceptibility to LC in a sample of 600 subjects from North India [25, 26]. A notable association with LC, e.g., for the *SFRP4* variant rs1802073 (OR  = 3.19; 95% CI 1.81–5.63), was reported. Classification And Regression Tree (CART) analysis revealed an interaction of *DKK2* and *SFRP4* polymorphisms to be the best (off all investigated) predictors for LC; especially within smokers. They also reported to have identified several high-risk subgroups in smokers, e.g., characterised by *DKK2* (rs17037102/rs419558) and *Axin2* (rs9915936). A similar picture was observed in a sample of 270 subjects from Istanbul, Turkey [27]. A two-way interaction between *DKK*3 (rs3206824) and *SFRP4* (rs1802074) was found to be predictive of LC.

We aimed to assess a possible association of *AhR* pathway and *Wnt* signalling cascade with LC within the large-scale series of cases and controls of European descent hold by the International Lung Cancer Consortium (ILCCO)/Integrative analysis of Lung Cancer Etiology and Risk (INTEGRAL). To do this, we also evaluated the contribution of these genes to genetic prediction of LC as a complement to known LC-related markers.

## Methods

The work presented has been reviewed and approved by the ILCCO Steering Committee.

### Cases and controls

Phenotype and genotype data of 58,181 entries of the data repository of ILCCO were extracted. Details of the repository is described previously [4, 28]. QC control samples, individuals without information on smoking status or age, and samples of poor genotyping quality or sex discrepancies, were excluded. To avoid population stratification, this analysis is focused on European-ancestry population (defined as more than 95% probability of being of European descent). Fourteen thousand sixty-eight incident LC-cases and 12,390 cancer-free controls of European descent remained for analysis. Those genotyped with other genome-wide array in addition to OncoArray were separated to form an independent validation set (2nd validation set) of size (*n*  =  4359, including 2360 LC-cases and 1999 controls).

### Selected markers

For this investigation we extracted the genotypes of 113 genomic variants (markers) assigned to 58 genes, previously associated with the risk for LC in European decent people or one of its histological subtypes through a wide variety of approaches [4–11] or proxies thereof (called *LC-marker*), and 296 markers assigned to 7 genes involved in *Wnt* signalling and listed in Bahl et al. [25, 26] and Yilmaz et al. [27] (called *AhR*/*Wnt-marker*). Thus, we focused this analysis to genes previously investigated with respect to LC. Fifty of these 409 markers were eliminated before analysis due to a MAF  < 1% (minor allele frequency), or departure from HWE (Hardy–Weinberg equilibrium) in genotypes (unaffected *p*  < 10^–7^, affected *p*  < 10^–12^), or low imputation accuracy (info  < 0.8). Seventy-eight of the remaining *LC-markers* were genotyped with the OncoArray (44 thereof are proxy SNPs identified using LDlink [29]) and 32 had to be imputed. Two hundred and twenty one of the remaining *AhR*/*Wnt-markers* were genotyped and 28 have been imputed. A list of these markers extracted from ILCCO OncoArray repository is given in the Additional file 1.

### Association analysis

We first performed association analysis for each marker separately using the program PLINK [30, 31]. Crude (model 1) and adjusted odds ratios (ORs) were estimated along with 95%-confidence intervals within log-additive models. Sex, age and smoking status and the first 3 principal components (PCs) to adjust for population stratification (model 2); and in addition the 6 most significantly associated *LC-markers* (rs55781567, 15q25.1 *CHRNA5*; rs11780471, 8p21.2 *CHRNA2*; rs7705526, 5p15.33 *TERT*; rs56113850, 19q13.2 *CYP2A6*; rs71658797, 1p31.1 AK5; rs11571833, 13q13.1 *BRCA2*) (model 3) were included in adjusted models. ORs were estimated for overall LC, small cell LC (SCLC), squamous cell LC (SqCLC), adenocarcinoma LC (adenoLC), ever smokers, never smokers and individuals aged  ≤ 55 years (early onset LC) as subgroups. We generated QQ-plots for the *AhR*/*Wnt-markers* and estimated the genomic inflation factor *λ*. To account for multiple testing, genome-wide statistical significance was considered to correspond to a *p* value of 10^−7^ or lower, suggestive significance to a *p* value between 10^−5^ and 10^−7^ and nominal significance to a *p* value between 0.05 and 10^−5^.

### Logistic regression—predicting models with model selection

We fitted logistic regression models with variable selection to find appropriate polygenic risk scores (PRS) to predict the disease (LC) status (affected or unaffected). Any *AhR*/*Wnt-marker* or the *LC-marker* could be included in the model without preference. To avoid multi-collinearity we removed one of two SNPs in LD to another (*R*^2^  > 0.8, pruning). The remaining entered the models as potential predictors. We performed forward selection until the Bayesian information criterion (BIC, most stringent selection), the Akaike information criterion (AIC, less stringent selection, contains in general more predictors) or the sample size corrected AIC (AICC) indicate a best solution (and 10 more selection steps). The resulting PRSs are called BIC-, AIC- and AICC-scores. Note, that for the purpose of model building, the AIC-selection is asymptotically equivalent to cross-validation (CV) [32, 33]. To avoid overfitting, we assigned individuals to a training or a validation set (to build a score) and a testing set (to examine the score performance) with a 1/3 probability each. For comparison, we also generated a BIC^LC^-score with at least one marker, only allowing *LC-markers* to enter the model building. To compare the importance for LC prediction of the sets $g$ of *LC-maker*s and *AhR*/*Wnt-markers, respectively,* we contrasted the importance-values defined as ${I}_{g}={\sum }_{m\exists g}\left|{\beta }_{m}\right|\bullet {\mathrm{MAF}}_{m}$ for each score (${\mathrm{MAF}}_{m}$ the minor allele frequency and ${\beta }_{m}$ the logistic regression coefficient of marker $m$). The superiority of the AIC-scores over the BIC^LC^-score and the BIC-score was tested applying the nonparametric test of DeLong et al. [34] (1-sided) on AUCs of ROC (area under the receiver operation characteristic curve). In addition, a corresponding precision-recall plot was created for the SCLC.

### Decision trees

Decision trees were created to examine marker × marker interaction with respect to the LC prediction. Any *AhR*/*Wnt-marker* or the *LC-marker* could be included in a tree without preference. This was accomplished in the entire sample and in all subgroups defined above. The R packages rpart and DescTools were used [35, 36]. To avoid trees being formed by spurious epistasis we removed one of two SNPs in LD to another (*R*^2^  > 0.8, pruning). Since overfitting is a point of concern when building decision trees, the complexity parameter was first optimized applying ten-fold cross-validation, grading the performance on the validation set by Somers’ D (concordance of true and predicted LC-status). The ability of the optimal trees to predict the LC-status was then tested within the independent sample of 4359 cases and controls. True positive (TP) and true negative (TN) rates are given.

All statistical analyses were performed with SAS^®^ 9.4, PLINK 1.90 and 2.0 or R 4.0.2.

### Gene expression

We extracted information on gene expression from the Human Protein Atlas [37, 38] and LungGENS [39, 40].

## Results

### Sample description

The analysed sample consists of 14,068 LC-cases and 12,390 controls with median age of 63. Sixty-three percent were male, 52% of cases and 28% of controls were current smokers. The most frequent histological subtype is adenocarcinoma (38%), followed by squamous cell carcinoma (SqCLC) (26%) and small cell lung cancer (SCLC) (10%). The proportion of never-smokers was largest within the subgroup of adenocarcinoma cases (14%), but almost the same between those cases aged  ≤ 55 years (10%) and aged  > 55 years (9%). Details on smoking status and histological subtypes are presented in Table 1.

**Table 1:** Smoking by LC status and subgroups

			Never smoker		Ever smoker					
		Total	Never		Former		Current		Ever^a^	
		*n*	*n*	%	*n*	%	*n*	%	*n*	%
Control										
Age ≤ 55 years		2762	951	34	698	25	896	32	217	8
Age > 55 years		9628	2960	31	3572	37	2568	27	528	5
All		12,390	3911	32	4270	34	3464	28	745	6
Case										
SqCLC	3692		138	4	1257	34	2158	58	139	4
SCLC	1450		48	3	383	26	965	67	54	4
Other LC	3629		405	11	1200	33	1820	50	204	6
AdenoLC	5297		740	14	1989	38	2401	45	167	3
Age ≤ 55 years	2765		281	10	452	16	1945	70	87	3
Age > 55 years	11,303		1050	9	4377	39	5399	48	477	4
All	14,068		1331	9	4829	34	7344	52	564	4
Total	26,458		5242	20	9099	34	10,808	41	1309	5

*SCLC* small cell lung cancer; *SqCLC* squamous cell lung cancer; *AdenoLC* adenocarcinoma of the lung; *other LC* other histological subtypes

^a^As recorded

### Association analysis

**Fig. 1:**
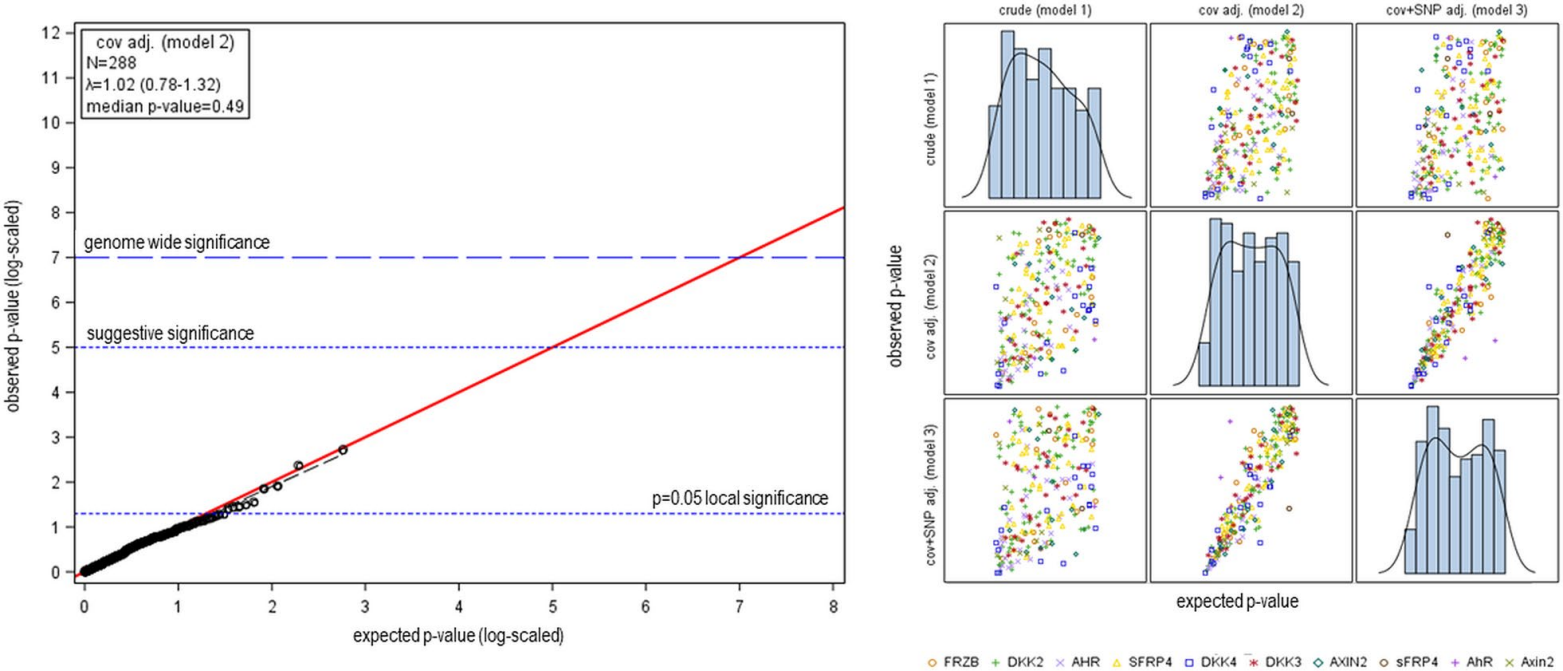
Association of *AhR*/*Wnt-marker.* Left panel: QQ-Plot for model 2 (adjusted for sex, age and smoking status and the first three principal components); right panel: matrix of *p* values generated by model 1 (crude), model 2 (adjusted for sex, age and smoking status and the first three principal components) and model 3 (additionally adjusted for 6 selected *LC-markers*), genome-wide significance: *p* value  ≤ 10^−7^, suggestive significance: 10^−7^  < *p* value  ≤ 10^−5^, nominal significance: 10^−5^  <  *p *value  ≤  0.05

We first performed association analysis for each *Wnt*/*AhR-marker* separately. The *p* values for an association of *AhR*/*Wnt-markers* with LC range from 0.005 (rs12115174; 8p11.21 *DKK4*; OR  = 0.9211) to 1 (model 2; adjusted for sex, age, smoking status and population stratification); with a negligible genomic inflation (*λ*  = 1.02). A nominally significant association (10^–5^  <  *p*  ≤  0.05) was observed for only 8 of the 249 markers (~ 3%). The corresponding point estimates of OR range from 0.88 (rs1053070054; 8p11.21 *DKK4*; *p*  = 0.007) to 1.12 (rs74596148; 7p14.1 *SFRP4*; *p*  = 0.25). A QQ-plot indicates that achieved *p* values almost perfectly agree with the expectation of no associated marker (see Fig. 1). *p* values and OR are in moderate agreement between the models (e.g., model 2–3; additionally adjusted by *LC-markers*: Kendall’s rho_p_  = 0.75, rho_OR_  = 0.78).

#### Subgroup analysis

When dividing the cases according to histological subtypes (SCLC; SqCLC and adenoLC) the observation of no detectable association for *WNT*/*AhR-markers* remains. Merely the number of nominally significant association (10^–5^  <  *p*  ≤ 0.05) increases to 12 (5%) or 21 (8%) of the 249 markers for SqCLC and SCLC, respectively, hence close to the expected type 1 error. (Additional file 1: Table S2). When dividing the cases and controls according to their smoking behaviour (ever and never smokers), genome-wide significance (*p*  ≤ 10^–7^) was achieved for 7 and 8 markers, respectively. Another 12 and 3 markers, respectively, were found suggestively significant (10^–7^  < *p*  ≤ 10^–5^) (see Additional file 1: Figure S1) for ever and never smokers. Those markers found associated among ever smokers have mainly been directly genotyped and are assigned to *SFRP4* and *DKK4*. For example, for marker rs2722278 we estimated an OR  = 1.20 (95% CI 1.13–1.27), yielding a *p* value of 5.6 × 10^–10^. Those markers found associated among never smokers have mainly been imputed and are mostly assigned to *Axin2,* but also to *AHR*, *FRZB* and *DKK2*. Marker rs17037102, assigned to *DKK2*, was the only one found associated with LC by Bahl et al. and in this analysis (see Table 2 and Additional file 1: Table S3). Interestingly, the ORs of these markers estimated by model 3 (additionally adjusted for selected *LC-marker*) differ from that estimated by model 2. They are closer to one and no more significant. For example, for rs1133683 (*Axin2*) we observe an OR  = 1.27 (95% CI 1.19–1.35, *p*  = 1 × 10^–12^) fitting model 2, but OR  = 0.95 (95% CI 0.86–1.06, *p*  = 0.3586) fitting model 3.

**Table 2:** Significantly associated *AhR*/*Wnt-markers* within never and ever smokers

SNP	Cyto band	MAF (%)	Gene	Model 2			Model 1	Model 3
				*p* value	OR	95% CI	OR	OR
Never smoker								
Imputed								
rs202198518^a^	7p21.1	14	*AHR*	3.4 × 10^–13^	0.72	0.66–0.79	0.71	0.90^ ns^
Imputed								
rs2237297^a^		14		9.9 × 10^–14^	0.71	0.65–0.78	0.71	0.90^ ns^
Imputed								
rs1133683	17q24.1	42	*Axin2*	1.0 × 10^–12^	1.27	1.19–1.35	1.27	0.95^ ns^
Imputed								
rs2240307		5		7.7 × 10^–24^	0.41	0.34–0.49	0.40	0.62^ ns^
Imputed								
rs35285779^a^		9		3.2 × 10^–22^	0.58	0.52–0.65	0.58	1.10^ ns^
Imputed								
rs35415678^a^		9		3.7 × 10^–19^	0.62	0.56–0.69	0.62	1.10^ ns^
Imputed								
rs288326	2q32.1	10	*FRZB*	2.5 × 10^–8^	1.42	1.25–1.60	1.41	0.98^ ns^
Imputed								
rs17037102	4q25	15	*DKK2*	7.4 × 10^–15^	0.69	0.63–0.76	0.69	1.09^ ns^
Ever smoker								
Genotyped								
rs12532321	7p14.1	45	*SFRP4*	1.3 × 10^–9^	1.14	1.09–1.19	1.15	1.13^ss^
Genotyped								
rs7811872		36		1.3 × 10^–8^	0.88	0.84–0.92	0.88	0.88^gws^
Genotyped								
rs10226308		42		1.8 × 10^–8^	0.88	0.85–0.92	0.89	0.89^gws^
Genotyped								
rs10488617		42		1.6 × 10^–8^	0.88	0.85–0.92	0.89	0.89^gws^
Genotyped								
rs2722278		16		5.6 × 10^–10^	1.20	1.13–1.27	1.16	1.20^gws^
Genotyped								
rs2722279		11		9.0 × 10^–9^	1.22	1.14–1.31	1.17	1.23^gws^
Genotyped								
rs7811420		43		7.9 × 10^–8^	0.89	0.85–0.93	0.89	0.89^gws^
Imputed								
rs2073664	8p11.21	9	*DKK4*	9.4 × 10^–11^	1.20	1.14–1.27	1.15	1.08^ss^

Model 1: crude odds ratio (OR); model 2: adjusted for sex, age and smoking status and the first three principal components; model 3: OR additional adjusted for 6 selected *LC-markers*. Only markers are listed for which genome-wide significance (*p* value  ≤ 10^–7^) was achieved

*MAF* minor allele frequency; ^*gws*^ genome-wide significant (*p* value  ≤ 10^−7^); ^*ss*^ suggestive significant (10^−7^  < *p* value  ≤ 10^−5^); ^*ns*^ not significant (*p*  > 0.05)

^a^Pair of markers in LD (*R*^2^  > 0.8 in populations of European decent)

### Logistic regression—predicting models with model selection

**Fig. 2:**
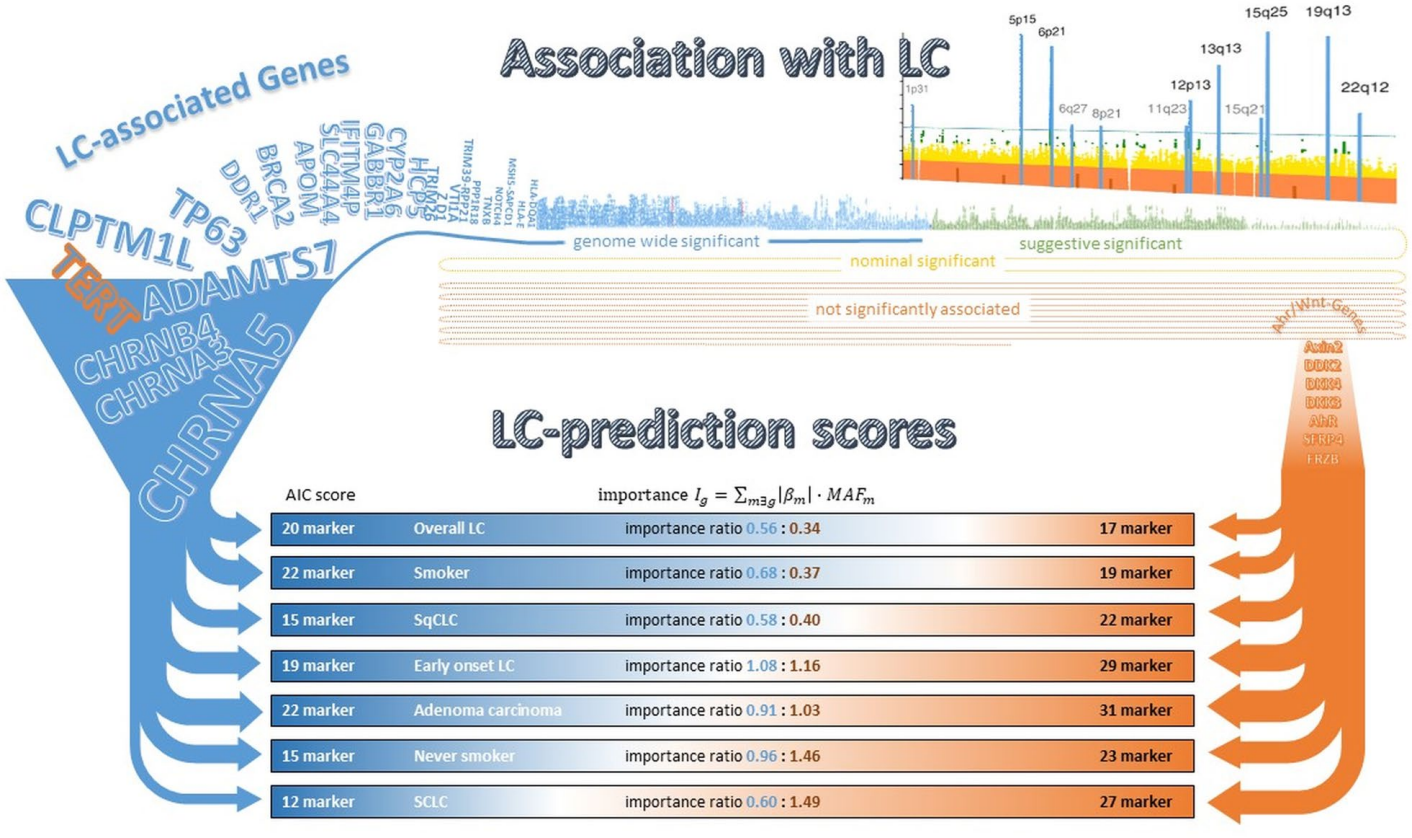
Comparison of score composition. *LC* lung cancer; *AIC score* score of a logistic regression model with variant selection according to the Akaike information criterion (AIC); *MAF*_*m*_ minor allele frequency of variant (marker) m; *β*_*m*_ regression parameter of variant m; *LC-associated genes* previously reported as associated to LC or one of its histological subtypes; *AhR*/*Wnt*-genes selected genes assigned to Wnt-signalling, including AhR; *Smoker* ever, former and current smoker; *SCLC* small cell lung cancer, *SqCLC* squamous cell lung cancer, Early onset LC: aged  ≤ 55 years; TERT is framed in orange, because telomerase activity is related to Wnt signalling

We further fit logistic regression models with variable selection to evaluate the contribution of *AhR*/*Wnt-markers* to a polygenic risk scores (PRS), but without postulating the usefulness of the score as such. Eight *LC-markers* from only eight *LC-genes* (*CYP2A6*, *CHRNA5*, *TERT*, *AMICA1*, *CHRNA3*, COPS2, *HCG4* and *CHRNA2*) were selected for the BIC-score (most stringent selection) to predict overall LC. Hence, the BIC-score and the BIC^LC^-score are identical. In contrast, the AIC-score (for overall LC identical to the AICC-score) includes 20 *LC-markers* and remarkable 17 *AhR*/*Wnt-markers*, with *LC-markers* being more important than the *AhR*/*Wnt-markers* (importance ratio 0.56: 0.34) (see Fig. 2, Additional file 1: Figure S3 and Table S4). The ability to distinguish cases and controls from susceptibility genes only was, as expected, poor for each of the scores (see Additional file 1: Table S5). In the training set the performance of the AIC/AICC-score (AUC  = 0.607) exceeded those of the BIC/BIC^LC^-score (AUC  = 0.582) significantly (*p*  < 0.001). Within the test set (AUCs: 0.577 and 0.576) and the 2nd validation set (AUCs: 0.553 and 0.548), the higher complexity with additional *AhR*/*Wnt-markers* did not improve discriminability for overall LC (*p*  = 0.87 and *p*  = 0.35).

Similar score composition and performance was observed for most subgroups. The BIC-scores in the subgroups adenoLC (involved marker LC:*AhR*/*Wnt* = 6:–), SCLC (3:–) and smokers (7:–) contained *LC-markers* only, whereas *AhR*/*Wnt-markers* are included even under this stringent variable selection in the subgroups SqCLC (5:1) and Early onset LC (2:2). However, between 14 and 31 *AhR*/*Wnt-markers* entered these subgroup’s AIC-scores. For these subgroups, the importance of the *LC-markers* for the AIC-score is higher than that of the included *AhR*/*Wnt-markers*.

**Fig. 3:**
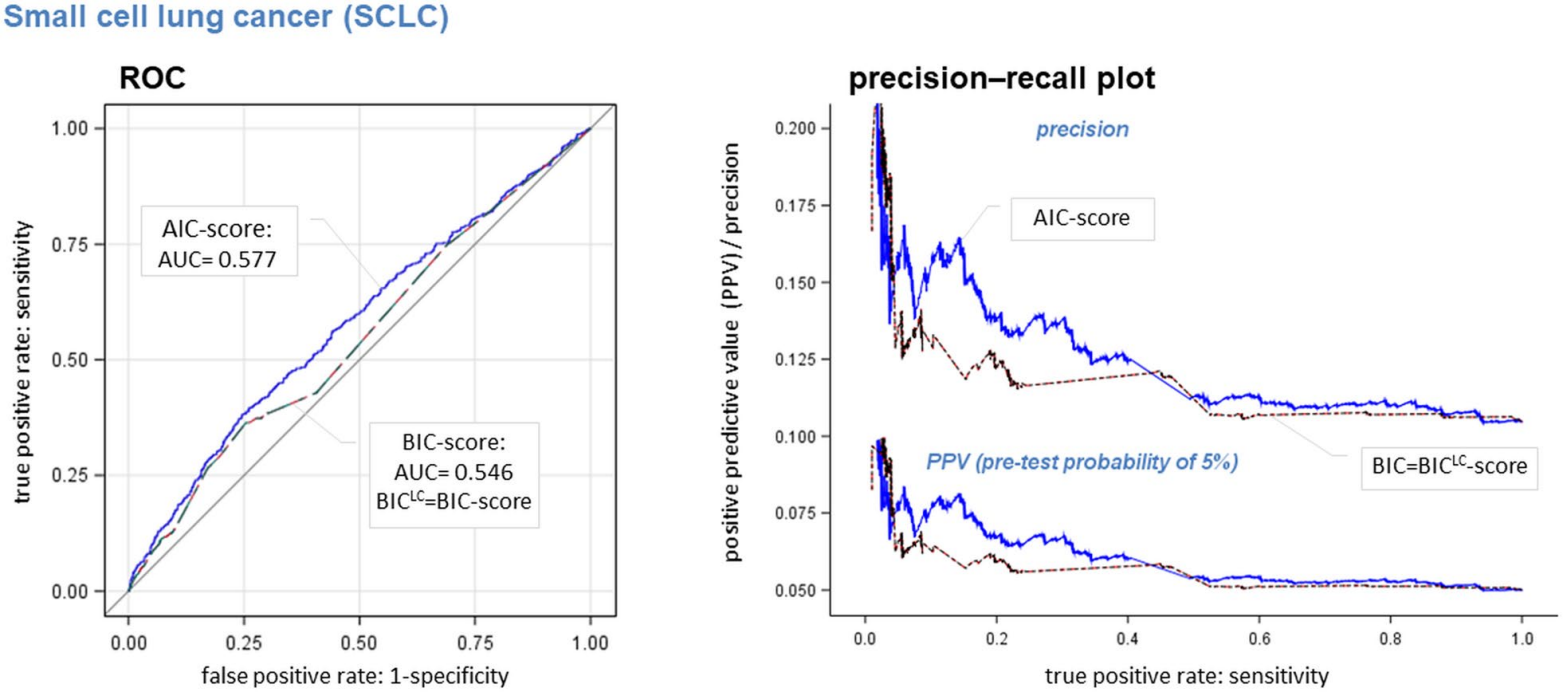
ROC and precision-recall-plot: SCLC. The diagnostic performance of the AIC-score compared to the BIC/BIC^LC^-score in the test-set is presented. Left panel: *ROC* receiver operation characteristics; right panel: corresponding precision-recall plot; precision  =  (true positive cases)/(true positive cases  +  false positive controls), positive predictive value (PPV)  =  (sensitivity × pre-test-probability)/[(sensitivity × pre-test-probability)  +  (1 − specificity  ×  1 − pre-test-probability)] for a pre-test-probability of 5%

Most important, we observed a significantly higher predictive accuracy (larger AUCs) of the *AhR*/*Wnt-markers* enriched AIC-scores compared to BIC^LC^–score in the subgroup of SCLC patients (*p*  = 0.019; AUC_AIC_  = 0.577 AUC_BIC_  = 0.546) within the test set (see Additional file 1: Figure S4). For this subgroup, the selected *AhR*/*Wnt-markers* contribute to the AIC-score more than twice as much as the *LC-markers* (importance ratio 0.60:1.49). The precision-recall plot of Fig. 3 indicates that a positive SCLC prediction based on the AIC-score can be trusted more than that based on *LC-markers* alone (BIC^LC^-score). In the 2nd validation set the score-specific AUCs were similar but no more significantly different (*p*  = 0.08; AUC_AIC_  = 0.564 vs. AUC_BIC_  = 0.531). The AIC-score of this SCLC-subgroup is composed of 12 *LC-markers* (assigned to *CHRNA5*, *HCG4*, *DNAJB4* (4 ×  each), *CYP2A6*, *CHRNA3*, *CHRNA2*, *AMICA1*, *KCNJ4*, *AS1*, *BRCA2*, *EGFL8* and *WNK1* (2 × each) and 27 *AhR*/*Wnt-markers* (assigned to all *AhR*/*Wnt*-genes except *DKK3*). However, only one LC patient in the test set (*n*  = 434) and one in the 2nd validation set (*n*  = 164) was recognized as a patient at a threshold of 50% case probability.

Interestingly the BIC-score for never smokers was built by only two *AhR*/*Wnt-markers* (assigned to *Axin2* and *SFRP4*) but not a single *LC-marker*. Furthermore, the *LC-markers* are the minority in the composite of the AIC-score (15:23). They also contribute less to the AIC-score than the *AhR*/*Wnt-markers* (importance ratio of 0.96:1.46). The median predicted case probability, in the test set (24.8%) and 2nd validation set (25.6%), exceeds that of controls by 1–2%-points. However, AUC differed neither in the test set (*p*  = 0.13; AUC_AIC_  = 0.540 AUC_BIC_  = 0.514) nor in the 2nd validation set (*p*  = 0.36; AUC_AIC_  = 0.535 AUC_BIC_  = 0.526) significantly. Nevertheless, this observation highlights the value of the *AhR*/*Wnt-markers* in the subgroup of never smokers.

### Decision trees

Finally, we generated decision trees to evaluate the contribution of *AhR*/*Wnt-markers* to LC prediction that allow for a complex interaction structure, but without postulating the usefulness of the trees as such. The decision tree for overall LC (whole sample) consists off solely a single decision node (rs55781567 assigned to *CHRNA5*), achieving a Somers’ concordance index *D*  = 0.0565 in the 2nd validation set (see Additional file 1: Table S6 and Figure S2). A single-node decision-tree was also found optimal for participants aged  ≤ 55 years (split: rs1051730 assigned to *CHRNA3*), achieving a Somers’ concordance index *D*  = 0.096. These two, unsophisticated trees are characterised by balanced TP- (about 62%) and TN-rates (about 44%).

The decision trees for ever smokers, SCLC and SqCLC were more complex achieving Somers’ concordance indexes *D* of 0.007, − 0.0005 and 0.0126, respectively. The trees for SCLC and SqCLC are characterised by an extreme TP-rate  < 5% and TN-rate  > 99%; the tree for Ever Smokers by a TP-rate  > 99% and TN-rate  < 5%. Remarkably, a marker assigned to *CHRNA5* was always chosen as the first and most important split for the trees for ever smokers, for SCC and SqCLC. However, markers assigned to *AhR*/*Wnt-genes* (smoker: *DKK2*; *SCLC*: *FRZB*; *SqCLC*; *DKK2* and *DKK3*) appear at lower-level decision-nodes (Additional file 1: Figures S5–S8). With the same program settings, no decision tree could be created for adenocarcinoma.

**Fig. 4:**
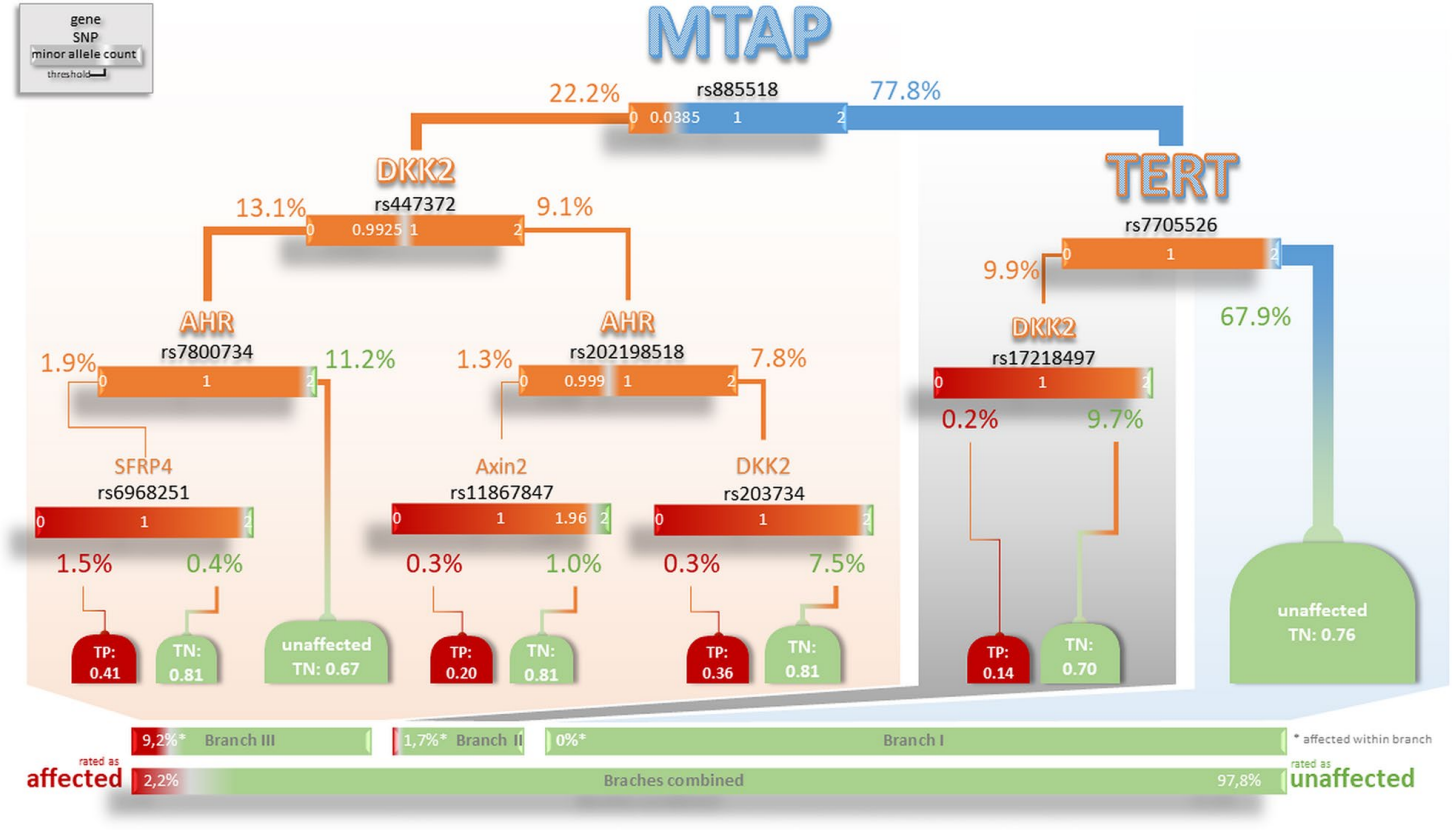
Decision tree for never smoker. Node information: gene name, marker; split information below the node: threshold for minor allele count; blue split nodes: LC-genes, orange split nodes: *AhR/Wnt*-genes,; TERT is framed in orange, because telomerase activity is related to Wnt signalling; decision nodes and bars: green for unaffected; red for affected, *TN* true negative rate, *TP* true positive red; the size of gene names, lines and decision notes is proportional to the size of the respective (sub)sample

Most notable is the optimal decision tree for the 5242 never smokers (75% LC-cases, 25% controls), the only one that does not contain a marker belonging to the CHRN (Cholinergic receptors nicotinic subunits) gene group (see Fig. 4). The tree is built from only two *LC-markers* but 7 *AhR*/*Wnt-markers*, achieving a Somers’ concordance index *D*  = − 0.002. One can make out three branches of this tree. Branch I covers two thirds of individuals (*n*  = 754, 66% of 1141 in the 2nd validation set): all of these are graded as “unaffected” based on only the two *LC-markers*: first decision node (rs885518 assigned to MTAP) and second decision node (rs7705526 assigned to TERT that links telomerase activity to Wnt signalling). For branch II an additional node (rs17214897 assigned to DKK2) is taken into account, covering a further tenth (9.9%) of never smokers. In this branch, very few subjects of the training set (1.7% within branch II eq. 0.17% of all never smokers) are graded “affected”. However, one in four individuals of the 2nd validation set belonging to both branches, I and II, is truly “affected” but has not been detected (TP-rate  = 0%, TN-rate  = 100%). Rated as “affected” appears in the test set only in the third branch III, covering the remaining fourth of never smokers (*n*  = 284 of the 2nd validation set). This third branch requires genotypes of several *AhR*/*Wnt-markers* assigned to *AHR*, *Axin2*, *DKK2* and/or *SFRP4*. Herein, one in three (*n*  = 97 of the 2nd validation set) is truly “affected” and is given a chance to be correctly identified, which appears in 8 LC-cases (TP-rate  = 9%, TN-rate  = 88%). We also noted that the histological subtypes are equally distributed between the branches (see Additional file 1: Table S7).

### Gene expression

*AHR*, *Axin2*, *DKK3* are ubiquitously expressed, with RNA expression detected in many tissues and evidence for protein expression. *Axin2* and *DKK3* are moderately to highly expressed in normal lung tissues according to the Human Protein Atlas [37]. *AhR* is expressed at low levels in macrophage cells of the lung. No expression is reported for other *Wnt*/*AhR-*genes (see Additional file 1: Figure S9 and Table S9). Significant differential expression is listed in LungGENS for *AhR*, *Axin2 DKK2*, *DKK3* and *SFRP4 *[39] (see Additional file 1: Table S8). Furthermore, AhR is reported to be abundantly expressed in solid lung tumours, especially in adenocarcinomas. AhR overexpression was associated with upregulation of IL-6 secretion, which is critical for lung cancer initiation [41]. Detailed information on gene expression is given in the Additional file 1. In addition, the *DKK*1 serum level was seen as significantly lower in NSCLC and SCLC patients compared to healthy controls [42]. Significant upregulation of *DKK2* expression was found in APC (adenomatous polyposis coli)-mutated non-SCLC lung cancers [43].

## Discussion

This investigation was intended to discover association of the *Wnt-*genes *DKK4* (8p11.21), *DKK3* (11p15.3), *DKK2* (4q25), *FRZB* (2q32.1, also known as *sFRP*3), *SFRP4* (7p14.1), *Axin2* (17q24.1) and a potential interaction with *AhR-genes,* to LC in a large sample of 26,458 individuals of European descent. No marginal association of *AhR*/*Wnt-markers* with overall LC was observed. Interestingly, an accumulation of associated markers was observed splitting the sample by smoking status, where respective markers in ever smokers are assigned to *SFRP4*. On the other hand, association analysis in never smokers reflects complex gene–gene interactions, as markers of several *AhR*/*Wnt-genes* were found to be genome-wide associated with LC. This complexity is also visible through the decision tree analysis.

Our results are in line with findings from northern India [25, 26] and from Istanbul, Turkey [27], both of which are based on much smaller samples (approx. 600 and 270 people, respectively). In these investigations, the interaction of DKK2 and DKK3 with SFRP4 and Axin2 polymorphisms turned out to be the best (of all examined) predictors of LC, especially in smokers. Axin2, but also AHR, FRZB and DKK2, were observed to be complex associated in never smokers. Our analysis agrees with both previous studies that complex interaction patterns between the examined genes contribute to overall LC susceptibility or within certain subgroups. However, we have not been able to replicate reported single marker associations directly.

To discover patterns of *AhR*/*Wnt-genes* involved in LC genesis we further changed the focus from significance of association to inclusion in prediction models, and followed two approaches: first, we searched for polygenic risk scores (PRS). Doing so, we add up marker main effects to construct multidimensional scores, optimising model fit (instead of marker preselection by p-value below some threshold), to discriminate cases from controls in a somehow ideal way. Complex gene × gene (G × G) interactions are not modelled.

Nevertheless, the proportion of *AhR*/*Wnt-genes* entering some of the predictive models was remarkable large, given that these markers are not, all other candidates, however, genome-wide significantly associated to LC. This was particularly noticeable for SCLC, since *AhR*/*Wnt-markers* contribute more than twice as much to the prediction score as *LC-markers*. It is known, that within current smokers, tobacco consumption is strongest associated to SCLC [44]. Moreover, within never smokers, a stringed defined score is made up from only two *AhR*/*Wnt-markers*, assigned to *Axin2* and *SFRP4*. However, the discriminative ability of PRSs for LC, contributing markers with significance for main effect at different levels, is in general poor. The AUC of the BIC^LC^ score for overall LC (0.58 in the test set and 0.55 in the 2nd validation set) corresponds to the AUC  = 0.54 based on four top *LC-genes* in a simulated population, as given by the GWAS-ROCS Database (https://gwasrocs.ca/). This may be due to other overpowering risk factors, since models including, e.g., age, sex and smoking variables achieve higher AUCs (0.62–0.79) [45].

Recently two polygenic risk scores (PRSs) for overall-LC had been developed, validated and assessed with respect to improving eligibility to low-dose computed tomography (LDCT) as the only recommended screening test for lung cancer. Jia et al. [46, 47] build a PRS on 19 genome-wide associated SNPs (*p*  < 0.5 × 10^–8^). Hung et al. [48], integrated their PRS on 128 SNPs (35 “known” LC-related loci, 93 suggestive associated loci selected by LASSO-regression model) into the PLCO_all2014_ risk model. Both approaches have been validated using data from the UK Biobank. For both scores, the mean PRS differed only slightly between LC cases and cancer-free controls (Jia: effect size  ~ 0.19; Hung: effect size  ~ 0.22). For both scores, no substantial increase in discriminability of cases from controls is reported, when adding the PRS to existing risk models (Jia: family history—AUC  = 0.589, family history  +  PRS – AUC  = 0.615; Hung: PLCO_all2014_–AUC  = 0.828, PLCO_all2014_  +  PRS – AUC  = 0.832). However, both were able to show that the age at which a smoker crosses the recommended screening threshold of 1.5% for the 5-year LC risk depends on the genetic background, which is sufficiently quantified by the PRS examined. Some smokers will be eligible by  < 50 years of age, others by  > 60 years of age. Hence, constructing reliable PRS, even with small discriminability, may help to improve the performance of LDCT.

Two- and multiway G × G interaction can also contribute to LC susceptibility, rather than just markers with observed (marginal) main effects. G × G interaction is in general less commonly investigated, not only because this requires much larger samples. However, Li et al. [49] found RGL1:RAD51B in overall LC and non-SCLC, SYNE1:RNF43 in adenocarcinoma and FHIT:TSPAN8 in SqCLC to interactively contribute to LC susceptibility. As in the presented data analysis, the impact of these genes would also have been overlooked considering main effects only. Another reason could be that LC itself is just a generic term of several subcategories that differ in terms of LC initiation and require separate PRSs [45, 50]. A third reason of the poor performance may be due to the exclusively concentration on genetic effects, rather than modelling lifelong interaction with the environment as well. For example, G × E interaction effects for LC have been observed smoking [51], exposure to asbestos fibres [52, 53] and exposure to radon [54, 55].

With this in mind, the data analysis presented shows that the complex interaction of Wnt-related genes has the potential to be part of an adequate risk assessment for never-smokers or in relation to certain histological subtypes of LC.

As a second approach, we constructed decision trees, which mainly depict G × G interaction patterns. Although, the ability to discriminate cases from controls is again poor, CHRNA5 was in general the most important first node for overall LC and in many subgroups. *AhR*/*Wnt-genes* play a complex but important role in at least one quarter of never smokers, as seen before. Remarkably, *TERT*, which links telomerase activity to *Wnt* signalling, was central in that branch and important for the remaining three quarters of never smoker. This corresponds to a concentration of relevant genes for this subgroup in the *CLPTM1L-TERT* region on chromosome 5, as previously reported by Hung et al. [56]. Our observations confirm the suspicion, that LC in never smokers is a different entity, justified beforehand on differences in epidemiological, clinical and molecular characteristics [50].

We would like to emphasize that this study was not intended to provide a definitive and reliable risk assessment, but rather aimed to examine in depth the LC-relevant complex interaction pattern of *AhR*/*Wnt-genes* hypnotized by Bahl et al. Indeed, considering prediction instead of association provides weaker evidence for this, but is valid in view of the large amount of external evidence. The importance of the *Wnt-*signalling pathway and its antagonist’s *sFRP*, *DKKs* and *A*xin*2* for cancer is outlined in the introduction. One can also assume a connection with the molecular functionality, since involved genes are expressed ubiquitously or in lung tissues.

Although the large-scale, thoroughly quality checked, and representative sample of genetically proven European descent individuals was used for the presented analysis, some limitations must be noted. We used a rather narrow definition of *AhR*/*Wnt-genes* to limit the number of possible interactions. An extension to, e.g., EGRF, APC, FRAT2 or the CYP-family would also be justified. We further could have chosen the random forest method as a more contemporary and robust approach than decision trees, but we would not be able to present our results so illustrative. However, the sample size allowed subgroup analyses, whereby the special importance of *AhR*/*Wnt-genes* for SCLC and never smokers could be shown.

## Conclusions

The role of markers belonging to *Wnt* signalling and the *AhR* pathway in LC susceptibility may be underrated in main-effects association analysis. Complex interaction patterns in individuals of European decent have moderate predictive capacity for LC or subsets thereof, especially in never smokers.

## Supplementary Information

**Additional file 1: Table S1.** List of investigated SNPs (*AhR/Wnt-markers* and *LC-markers*). **Table S2.** Association of *AhR/Wnt-marker *within subgroups. **Table S3.** Association of markers reported elsewhere. **Table S4.** Score composition and importance ratio. **Table S5.** Discriminability of prediction scores. **Table S6.** Prediction accuracy of the decision trees. **Table S7.** Prediction accuracy in never smokers by histological subtypes. **Table S8.** Expression in normal tissue of the lung according LungGENS. **Table S9.** Expression in normal tissue of the lung according the Human Protein Atlas. **Figure S1.** Association of *AhR/Wnt-markers *within never and ever smokers. **Figure S2.** Decision tree for overall LC. **Figure S3.** LC-risk score: model selection and ROCs for overall LC. **Figure S4.** LC-risk score: model selection and ROCs for SCLC and Never smoker. **Figure S5.** Decision tree for early onset LC (age ≤55 years). **Figure S6.** Decision tree for SqCLC. **Figure S7.** Decision tree for SCLC. **Figure S8.** Decision tree for ever smoker. **Figure S9.** Expression profiles according to the Human Protein Atlas.

## Data Availability

The data that support the findings of this study are available from ILCCO/INTEGRAL but restrictions apply to the availability of these data, which were used under license for the current study, and so are not publicly available. Data are, however, available from the authors upon reasonable request and with permission of ILCCO/INTEGRAL.

## Abbreviations

AhR: Aryl hydrocarbon receptor
GWAS: Genome-wide association studies
LC: Lung cancer
NSCLC: Non-small cell lung cancer
SCLC: Small cell lung cancer
SqCLC: Squamous cell lung cancer
adenoLC: Adenocarcinoma lung cancer
OR: Odds ratio
CART: Classification and regression tree
AUCs of ROC: Area under the receiver operation characteristic curve
ILCCO: International Lung Cancer Consortium
INTEGRAL: Integrative analysis of lung cancer etiology and risk
PRS: Polygenic risk scores
BIC: Bayesian information criterion
AIC: Akaike information criterion
CV: Cross validation
MAF: Minor allele frequency
TP: True positive rate
TN: True negative rate
LDCT: Low-dose computed tomography
