# Supplementary material for: Gene–gene interaction of AhRwith and within the Wntcascade affects susceptibility to lung cancer
Source: Eur J Med Res. 2022 Jan 31;27:14. doi: 10.1186/s40001-022-00638-7 (PMC8805279; doi:10.1186/s40001-022-00638-7)
Supplement: Supplementary file 1 — Additional file 1: Table S1. List of investigated SNPs (AhR/Wnt-markers and LC-markers). Table S2. Association of AhR/Wnt-marker within subgroups. Table S3. Association of markers reported elsewhere. Table S4. Score composition and importance ratio. Table S5. Discriminability of prediction scores. Table S6. Prediction accuracy of the decision trees. Table S7. Prediction accuracy in never smokers by histological subtypes. Table S8. Expression in normal tissue of the lung according LungGENS. Table S9. Expression in normal tissue of the lung according the Human Protein Atlas. Figure S1. Association of AhR/Wnt-markers within never and ever smokers. Figure S2. Decision tree for overall LC. Figure S3. LC-risk score: model selection and ROCs for overall LC. Figure S4. LC-risk score: model selection and ROCs for SCLC and Never smoker. Figure S5. Decision tree for early onset LC (age ≤55 years). Figure S6. Decision tree for SqCLC. Figure S7. Decision tree for SCLC. Figure S8. Decision tree for ever smoker. Figure S9. Expression profiles according to the Human Protein Atlas. [file 40001_2022_638_MOESM1_ESM.pdf]

Gene-gene interaction of AhR with and within the Wnt cascade affects susceptibility to lung cancer.

European Journal of Medical Research.

Albert Rosenberger et al. on behalf of the INTEGRAL-ILCCO consortium

Corresponding author: Albert Rosenberger, PhD
Universitätsmedizin Göttingen, Institut für Genetische Epidemiologie
Humboldtallee 32, 37073 Göttingen, Germany
Email: arosenb@gwdg.de

Supplementary material

Marker extracted from ILCCO OncoArray repository

The following markers were extracted from ILCCO OncoArray repository. Imputed genotype probabilities were converted to minor allele counts (ranging between 0 and 2). Marker names given by Bahl et al. 2017. [1, 2] were translated into rs-numbers according to: Leiden Open Variation Database (LOVD; https://databases.lovd.nl/shared/variants/) in combination with dbClinVAR (https://www.ncbi.nlm.nih.gov/clinvar/). Crossed out markers could not be extracted, and some were replaced by proxy-markers (according to LDLink [3]).

Ahr/Wnt-markers

We extracted all markers assigned to the following genes, as investigated by Bahl et al. 2017. [1, 2]:

- *DKK2* *rs447372, rs17037102 and rs419558: rs402002 rs402586 rs392056 (as proxy for rs419558)*
- *DKK3* *rs2291599, rs7396187 and rs3206824: rs13903 rs10831690 rs10741562 (as proxy for , rs3206824),*
  rs7391689: is monoallelic in EUR, located at CHR X - is not assigned to DKK3 (according to the Ensemble database)
- *DKK4* *rs2073664,*
- *sFRP3* *rs7775 (alternative name of rs52801397, rs3197283) , rs288326*
- *SFRP4* *rs1802073, rs1802074*
- *AhR* *rs10250822 rs7811989, rs2282885 and rs202198518 rs2074113 rs2237297 (as proxy for rs2066853)*
- *Axin2* *rs9915936 / 1365 G>A, rs1133683 / 1386 C>T, rs35415678 / 2062 C>T, rs2240307 / 432 T>C, rs35285779 / 956 + 16 A>G*
  and rs9903665 rs11867847 rs4791168 (as proxy for rs2240308 /148 C>T)

Investigated markers

S-Table 1 List of investigated SNPs (Ahr/Wnt-markers and LC-markers)

SNP	cytoBand	gene
rs71658797	1p31.1	*AK5*
rs34517439	1p31.1	*DNAJB4*
chr2_173983204_A_G	2q31.1	*MAP3K20*
rs1056836	2p22.2	*CYP1B1*
chr2_173763474_A_G	2q31.1	*MAP3K20*
rs2578007	2q32.1	*FRZB*
rs11887488	2q32.1	*FRZB*
chr2_183524090_C_T	2q32.1	*FRZB*
chr2_183534047_A_T	2q32.1	*FRZB*
chr2_183534216_C_T	2q32.1	*FRZB*
rs4322805	2q32.1	*FRZB*
chr2_183577930_G_T	2q32.1	*FRZB*
chr2_183582104_C_T	2q32.1	*FRZB*
rs11678585	2q32.1	*FRZB*
chr2_183623977_G_T	2q32.1	*FRZB*
rs6731059	2q32.1	*FRZB*
rs13509	2q32.1	*FRZB*
rs288298	2q32.1	*FRZB*
rs10931040	2q32.1	*FRZB*
rs288354	2q32.1	*FRZB*
chr2_183695449_A_G	2q32.1	*FRZB*
chr2_183698691_A_G	2q32.1	*FRZB*
chr2_183701014_A_T	2q32.1	*FRZB*
rs3768842	2q32.1	*FRZB*
rs2118588	2q32.1	*FRZB*
chr2_183710898_C_T	2q32.1	*FRZB*
rs6733333	2q32.1	*FRZB*
rs6433993	2q32.1	*FRZB*
rs12992123	2q32.1	*FRZB*
rs10202627	2q32.1	*FRZB*
chr2_183753187_C_T	2q32.1	*FRZB*
rs7636839	3q28	*TP63*
rs6448050	4p15.2	*KCNIP4*
rs10516537	4q25	*DKK2*
rs1443893	4q25	*DKK2*
rs17482054	4q25	*DKK2*
rs12508455	4q25	*DKK2*
rs13125118	4q25	*DKK2*
rs10014676	4q25	*DKK2*
kgp8163145	4q25	*DKK2*
chr4_107827275_A_G	4q25	*DKK2*
rs450533	4q25	*DKK2*
chr4_107835919_A_G	4q25	*DKK2*
rs10488898	4q25	*DKK2*
rs419558	4q25	*DKK2*
chr4_107845198_A_G	4q25	*DKK2*
chr4_107846365_G_INDEL	4q25	*DKK2*
exm417612	4q25	*DKK2*
rs433201	4q25	*DKK2*
rs10488899	4q25	*DKK2*
chr4_107870850_AGTC_INDEL	4q25	*DKK2*
chr4_107891481_A_G	4q25	*DKK2*
rs2051756	4q25	*DKK2*
rs78131467	4q25	*DKK2*
chr4_107913875_A_C	4q25	*DKK2*
chr4_107941676_G_T	4q25	*DKK2*
rs4956275	4q25	*DKK2*
rs2704335	4q25	*DKK2*
rs383567	4q25	*DKK2*
chr4_107964935_A_C	4q25	*DKK2*
rs7685856	4q25	*DKK2*
rs419783	4q25	*DKK2*
chr4_107978475_C_T	4q25	*DKK2*
rs2704324	4q25	*DKK2*
rs378405	4q25	*DKK2*
rs6849760	4q25	*DKK2*
rs403608	4q25	*DKK2*
rs81299	4q25	*DKK2*
rs382525	4q25	*DKK2*
rs435887	4q25	*DKK2*
rs17510289	4q25	*DKK2*
rs17510449	4q25	*DKK2*
rs2158182	4q25	*DKK2*
rs718820	4q25	*DKK2*
rs2522457	4q25	*DKK2*
rs409307	4q25	*DKK2*
rs28513708	4q25	*DKK2*
chr4_108072846_C_T	4q25	*DKK2*
rs6826864	4q25	*DKK2*
rs17037473	4q25	*DKK2*
rs17483781	4q25	*DKK2*
chr4_108117014_A_G	4q25	*DKK2*
rs1027253	4q25	*DKK2*
rs17549820	4q25	*DKK2*
rs2029635	4q25	*DKK2*
rs4403120	4q25	*DKK2*
rs17037525	4q25	*DKK2*

SNP	cytoBand	gene
rs11941336	4q25	*DKK2*
chr4_108156814_C_T	4q25	*DKK2*
chr4_108157443_C_T	4q25	*DKK2*
rs11722765	4q25	*DKK2*
rs78389285	4q25	*DKK2*
chr4_108177821_A_C	4q25	*DKK2*
chr4_108187241_A_G	4q25	*DKK2*
rs1038854	4q25	*DKK2*
rs72669703	4q25	*DKK2*
rs11946611	4q25	*DKK2*
rs1056079	4q25	*DKK2*
rs6533295	4q25	*DKK2*
rs717435	4q25	*DKK2*
rs203734	4q25	*DKK2*
rs1502773	4q25	*DKK2*
rs17218497	4q25	*DKK2*
rs203721	4q25	*DKK2*
rs6847720	4q25	*DKK2*
rs1502768	4q25	*DKK2*
rs203221	4q25	*DKK2*
rs203227	4q25	*DKK2*
rs203202	4q25	*DKK2*
chr4_108375341_C_T	4q25	*DKK2*
rs11722979	4q25	*DKK2*
rs2214389	4q25	*DKK2*
rs10005025	4q25	*DKK2*
rs17501731	4q25	*DKK2*
corect_rs115647190	4q25	*DKK2*
rs1399403	4q25	*DKK2*
rs6811222	4q25	*DKK2*
rs1913586	4q25	*DKK2*
rs2672469	4q25	*DKK2*
chr4_108469756_A_G	4q25	*DKK2*
rs7705526	5p15.33	*TERT*
rs2736100	5p15.33	*TERT*
rs2853677	5p15.33	*TERT*
rs4635969	5p15.33	*MIR4457*
rs61574973	5p15.33	*MIR4457*
rs4975616	5p15.33	*CLPTM1L*
rs421629	5p15.33	*CLPTM1L*
rs380286	5p15.33	*CLPTM1L*
rs402710	5p15.33	*CLPTM1L*
rs467095	5p15.33	*CLPTM1L*
rs40181	5p15.33	*CLPTM1L*
chr6_31081205_A_G	6p21.33	*C6orf15*
chr6_29660816_A_C	6p22.1	*HCG4*
rs34493019	6p22.2	*HIST1*
rs17598658	6p22.2	*HIST1*
chr6_26309908_A_G	6p22.2	*BTN3A2*
rs200484	6p22.1	*HIST1*
chr6_27815639_A_G	6p22.1	*HIST1H2BN*
rs188015	6p22.1	*HIST1H2BO*
rs13197574	6p22.1	*ZSCAN12P1*
rs34662244	6p22.1	*ZSCAN*
rs13213152	6p22.1	*ZKSCAN3*
rs1233385	6p22.1	*GABBR1*
rs2734986	6p22.1	*GABBR1*
rs3094222	6p21.33	*C6orf15*
rs3094604	6p21.33	*HCP5*
rs1800629	6p21.33	*LTA*
rs3132445	6p21.33	*MSH5*
rs3131378	6p21.33	*MSH5*
rs1270942	6p21.33	*C2*
rs3130288	6p21.32	*EGFL8*
rs2187668	6p21.32	*DQA1*
rs439553	6q27	*RNASET2*
rs3769201	6p21.33	*CSNK2B*
rs7604288	6p21.33	*CSNK2B*
rs6950788	7p21.1	*AHR*
rs4145933	7p21.1	*AHR*
rs4524681	7p21.1	*AHR*
corect_rs144861496	7p21.1	*AHR*
rs4391312	7p21.1	*AHR*
chr7_17265431_AT_INDEL	7p21.1	*AHR*
rs1476079	7p21.1	*AHR*
rs6968554	7p21.1	*AHR*
chr7_17287287_A_G	7p21.1	*AHR*
rs17137472	7p21.1	*AHR*
rs12670403	7p21.1	*AHR*
rs765623	7p21.1	*AHR*
rs3757824	7p21.1	*AHR*
rs10249788	7p21.1	*AHR*
rs10253202	7p21.1	*AHR*
rs12532771	7p21.1	*AHR*
chr7_17343594_A_G	7p21.1	*AHR*
rs17779352	7p21.1	*AHR*
rs17137566	7p21.1	*AHR*
rs6960165	7p21.1	*AHR*
chr7_17375302_C_T	7p21.1	*AHR*
rs2066853	7p21.1	*AHR*
kgp23389916	7p21.1	*AHR*
kgp13827077	7p21.1	*AHR*
rs35618592	7p21.1	*AHR*
kgp23662521	7p21.1	*AHR*
kgp13656381	7p21.1	*AHR*
rs2053998	7p21.1	*AHR*
rs77890351	7p21.1	*AHR*
corect_rs144144682	7p21.1	*AHR*
rs6947693	7p21.1	*AHR*
chr7_17439471_C_T (rs7800734)	7p21.1	*AHR*
rs6461315	7p21.1	*AHR*
chr7_17451588_A_G	7p21.1	*AHR*
rs4721602	7p21.1	*AHR*
rs4721604	7p21.1	*AHR*
rs12699853	7p21.1	*AHR*
rs2034684	7p21.1	*AHR*
chr7_37922589_A_G (rs12532321)	7p14.1	*SFRP4*
rs62001870	7p14.1	*SFRP4*
chr7_37935150_A_G (rs7811872)	7p14.1	*SFRP4*
rs2598119	7p14.1	*SFRP4*
rs10488617	7p14.1	*SFRP4*
rs10226308	7p14.1	*SFRP4*
rs10276139	7p14.1	*SFRP4*
rs7811420	7p14.1	*SFRP4*
rs1530820	7p14.1	*SFRP4*
rs1450857	7p14.1	*SFRP4*
rs1132552	7p14.1	*SFRP4*
rs1376264	7p14.1	*SFRP4*
rs6968251	7p14.1	*SFRP4*
rs1349394	7p14.1	*SFRP4*
rs3734952	7p14.1	*SFRP4*
chr7_37966845_A_T (rs74596148)	7p14.1	*SFRP4*
rs2598107	7p14.1	*SFRP4*
chr7_37977116_C_T	7p14.1	*SFRP4*
rs2167267	7p14.1	*SFRP4*
rs2044831	7p14.1	*SFRP4*
rs2722276	7p14.1	*SFRP4*
rs2722278	7p14.1	*SFRP4*
rs2722279	7p14.1	*SFRP4*
chr7_37991186_C_T	7p14.1	*SFRP4*
rs1450850	7p14.1	*SFRP4*
rs1621407	7p14.1	*SFRP4*
rs55821827	7p14.1	*SFRP4*
corect_rs145956937	7p14.1	*SFRP4*
rs1668349	7p14.1	*SFRP4*
rs1524053	7p14.1	*SFRP4*
chr7_38046212_C_T	7p14.1	*SFRP4*
rs34382627	7p14.1	*SFRP4*
rs1464717	7p14.1	*SFRP4*
rs1524078	7p14.1	*SFRP4*
rs1721400	7p14.1	*SFRP4*
rs17171260	7p14.1	*SFRP4*
rs1524072	7p14.1	*SFRP4*
rs1357653	7p14.1	*SFRP4*
rs1980313	7p14.1	*SFRP4*
rs7781364	7p14.1	*SFRP4*
rs1474346	7p14.1	*SFRP4*
rs1014842	7p14.1	*SFRP4*
rs940347	7p14.1	*SFRP4*
chr7_38121295_A_C	7p14.1	*SFRP4*
rs17408199	7p14.1	*SFRP4*
rs1524066	7p14.1	*SFRP4*
rs1524063	7p14.1	*SFRP4*
chr7_38145023_G_INDEL	7p14.1	*SFRP4*
rs11780471	8p21.2	*CHRNA2*
rs2439312	8p12	*NRG1*
rs12115174	8p11.21	*DKK4*
rs7829991	8p11.21	*DKK4*
chr8_42103378_A_G (rs1053070054)	8p11.21	*DKK4*
chr8_42125211_C_G	8p11.21	*DKK4*
rs11986055	8p11.21	*DKK4*
chr8_42135681_A_G	8p11.21	*DKK4*
rs5029748	8p11.21	*DKK4*
rs10099598	8p11.21	*DKK4*
rs2272733	8p11.21	*DKK4*
chr8_42177163_A_G	8p11.21	*DKK4*
rs10958713	8p11.21	*DKK4*
chr8_42187712_A_G	8p11.21	*DKK4*
chr8_42198512_A_G	8p11.21	*DKK4*
chr8_42207547_C_G	8p11.21	*DKK4*
chr8_42213073_A_G	8p11.21	*DKK4*
chr8_42226805_C_G	8p11.21	*DKK4*
rs10097867	8p11.21	*DKK4*
rs28510568	8p11.21	*DKK4*
chr8_42250447_A_G	8p11.21	*DKK4*
chr8_42254380_C_T	8p11.21	*DKK4*
rs3736649	8p11.21	*DKK4*
chr8_42268224_C_T	8p11.21	*DKK4*
rs3763510	8p11.21	*DKK4*
chr8_42292112_A_C	8p11.21	*DKK4*
corect_rs117922533	8p11.21	*DKK4*
chr8_42317097_C_T	8p11.21	*DKK4*
chr8_42339410_A_G	8p11.21	*DKK4*
rs7029287	9p21.3	*MTAP*
rs62560775	9p21.3	*AS1*
rs1333040	9p21.3	*CDKN2A*
rs9420907	10q24.33	*OBFC1*
rs4910417	11q23.3	*SCN2B*
chr11_11968234_A_G	11p15.3	*DKK3*
rs10741564	11p15.3	*DKK3*
rs2307073	11p15.3	*DKK3*
rs1979687	11p15.3	*DKK3*
rs3206824	11p15.3	*DKK3*
rs7396140	11p15.3	*DKK3*
chr11_12002564_C_G	11p15.3	*DKK3*
chr11_12004368_C_T	11p15.3	*DKK3*
rs4757595	11p15.3	*DKK3*
rs12295349	11p15.3	*DKK3*
chr11_12010327_A_C	11p15.3	*DKK3*
chr11_12010496_C_T	11p15.3	*DKK3*
rs6485350	11p15.3	*DKK3*
rs7116879	11p15.3	*DKK3*
rs12421658	11p15.3	*DKK3*
chr11_12020026_A_C	11p15.3	*DKK3*
chr11_12024576_G_T	11p15.3	*DKK3*
chr11_12025743_C_T	11p15.3	*DKK3*
rs4290212	11p15.3	*DKK3*
rs2896596	11p15.3	*DKK3*
rs903012	11p15.3	*DKK3*
rs4500466	11p15.3	*DKK3*
rs4307701	11p15.3	*DKK3*
rs923805	11p15.3	*DKK3*
rs7395308	11p15.3	*DKK3*
rs10501832	11q21	*MTMR2*
rs1056562	11q23.3	*AMICA1*
chr12_1072696_C_T	12p13.33	*WNK1*
chr13_32972626_A_T (rs11571833)	13q13.1	*BRCA2*
rs11571815	13q13.1	*BRCA2*
chr15_78806023_C_T	15q25.1	*HYKK*
chr15_78857986_C_G (rs55781567)	15q25.1	*CHRNA5*
chr15_78878541_A_G	15q25.1	*CHRNA5*
rs2640726	15q15.3	*TP53BP1*
rs690367	15q15.3	*TP53BP1*
rs1378214	15q21.1	*SEMA6D*
rs2413932	15q21.1	*COPS2*
rs34606419	15q21.2	*GALK2*
rs8040868	15q25.1	*CHRNA3*
rs17483929	15q25.1	*IREB2*
rs56117933	15q25.1	*PSMA4*
rs2036527	15q25.1	*CHRNA5*
chr15_78894339_A_G (rs1051730)	15q25.1	*CHRNA3*
rs12914385	15q25.1	*CHRNA3*
rs6495309	15q25.1	*CHRNA3*
rs10851907	15q25.1	*CHRNB4*
rs77719127	15q25.1	*ADAMTS7*
rs17244648	15q25.1	*CTSH*
rs12936580	17q24.1	*Axin2*
rs4386170	17q24.1	*Axin2*
rs4128941	17q24.1	*Axin2*
rs4541111	17q24.1	*Axin2*
chr17_63536373_A_G	17q24.1	*Axin2*
rs4791171	17q24.1	*Axin2*
chr17_63549261_C_T	17q24.1	*Axin2*
chr17_63549488_A_C	17q24.1	*Axin2*
chr17_63550241_C_T	17q24.1	*Axin2*
chr17_63550543_A_G	17q24.1	*Axin2*
chr17_63554591_A_G	17q24.1	*Axin2*
chr17_63559583_A_G	17q24.1	*Axin2*
rs8073426	17q24.1	*Axin2*
chr17_63565910_C_G	17q24.1	*Axin2*
rs1468329	17q24.1	*Axin2*
rs7216210	17q24.1	*Axin2*
chr17_63602081_C_T	17q24.1	*Axin2*
rs1350551	18q12.1	*GAREM1*
rs11670760	19q13.2	*CYP2A6*
rs56113850	19q13.2	*CYP2A6*
rs1709084	19q13.2	*CYP2A13*
rs3761121	20q13.33	*RTEL1*
chr22_29121087_A_G	22q12.1	*CHEK2*
rs17879961	22q12.1	*CHEK2*
rs111105	22q13.1	*KCNJ4*

Association

S-Table 2 Association of *AhR/Wnt-marker* within Subgroups

	p-value				significance				OR		
	N	min	median	max	genome-wide	suggestive	nominally	not sig.	min	median	max
**AdenoLC**	249	0.002	0.51	0.997	--	--	9	240	0.84	0.99	1.17
**SCLC**	249	0.0002	0.53	0.998	--	--	21	228	0.72	1.00	1.26
**SqCLC**	249	0.007	0.49	0.997	--	--	12	237	0.82	1.00	1.24
**ever smoker**	244	$9.4\ 10^{-11}$	0.33	0.996	7	12	36	189	0.48	1.00	3.80
**never smoker**	249	$7.7\ 10^{-24}$	0.45	0.998	8	3	15	223	0.41	0.99	1.62
**age ≤ 55 yrs.**	249	0.0005	0.47	0.999	--	--	9	240	0.80	1.00	1.54
**Overall LC**	249	0.002	0.50	0.997	--	--	7	242	0.88	1.00	1.12

N marker count, OR odds ratio; 95%-CI 95% confidence interval; min minimum, max maximum; LC: overall lung cancer, SCLC: small cell lung cancer, SqCLC : squamous cell lung cancer, AdenoLC: adenocarcinoma of the lung

S-Figure 1 Association of *AhR/Wnt-markers* within never and ever smokers

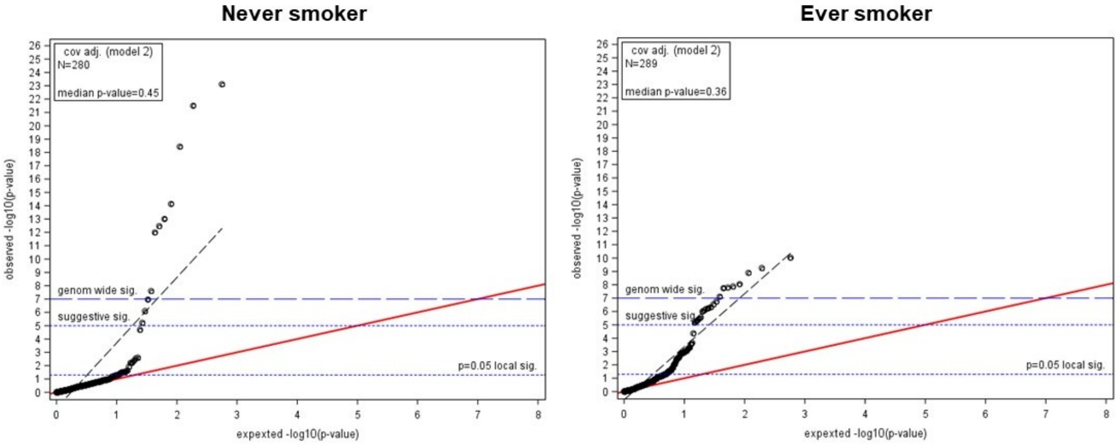

S-Table 3 Association of markers reported elsewhere

			Yilmaz et al. 2015			*Bahl et al. 2017*			*ILLCO/INTEGRAL sample*		
gene	*marker*		*OR[&]*	*95%-CI:*	*p-value*	*OR[§]*	*95%-CI*	*p-value*	*OR[$]*	*95%-CI*	*p-value*
DKK3	**rs3206824**	GA	0.20	0.02–1.52	0.12	1.14	0.79–1.64	0.47	0,99	0,94-1.04	0.80
	(ref. GG)	AA	0.08	0.01–0.70	0.02	1.29	0.38–4.39	0.68			
within never-smokers		GA	*n.r.*			1.91	0.93–3.89	0.04	0,97	0.86-1.10	0.63
DKK3	**rs7396187**	GC	*n.r.*			0.60	0.44–0.94	0.02	0.98	0.93-1.02	0.33
	(ref. GG)	CC	*n.r.*			*n.r.*					
within AdenoLC			*n.r.*			*0.45*	*0.26–0.78*	*0.0045*	0.98	0.93-1.03	0.33
DKK2	***rs17037102***	GA	1.57	0.08–28.8	0.76	*1.62*	*1.09–2.40*	*0.01*	1.03	0.96-1.10	0.38
	(ref. GG)	AA	2.64	0.13–51.5	0.52	*1.69[§§]*	*1.14–2.50*	*0.008*			
within never-smokers			*n.r.*			*2.69[§§]*	*1.16–6.24*	*0.02*	0.69	0.62-0.76	$7.4\ 10^{-15}$
DKK2	**rs419558**	CT	*n.r.*			*1.77*	*1.21–2.58*	*0.002*	1.02	0.97-1.07	0.79
	(ref. CC)	TT	*n.r.*			*n.r.*					
SFRP4	**rs1802074**	AA	2.40	0.54–10.5	0.24	1.74	1.07–2.83	0.02	0.99	0.96-1.04	0.94
	(ref. GG)	GA	0.91	0.47–1.74	0.77	1.66	0.66–4.18	0.27			

n.r. not reported; ref. reference genotype (homozygote major allele); OR odds ratio; 95%-CI 95% confidence interval; [&] adjusted for smoking, BMI and familial history; [§] adjusted for age, gender and smoking; [§§] GA+ AA; [$] per allele, adjusted for age, sex, smoking and 3 principal components (PCs) of a genome-wide set of marker to adjust for population stratification; AdenoLC: adenocarcinoma of the lung

Polygenic Risk Scores (PRS) and Decision Trees: Overall LC

S-Figure 2 Decision tree for overall LC

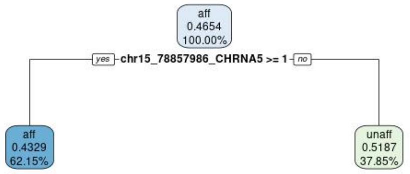

Node information: “unaff”=control (unaffected), “aff”=LC-case (affected), percentage of true decision, portion of sample rated at this node (sums to 100% across each level). Colour code refers to the decision: green=affected, blue=unaffected; split information below the node: marker, gene and threshold for minor allele count.

S-Figure 3 LC-risk score: model selection and ROCs for overall LC

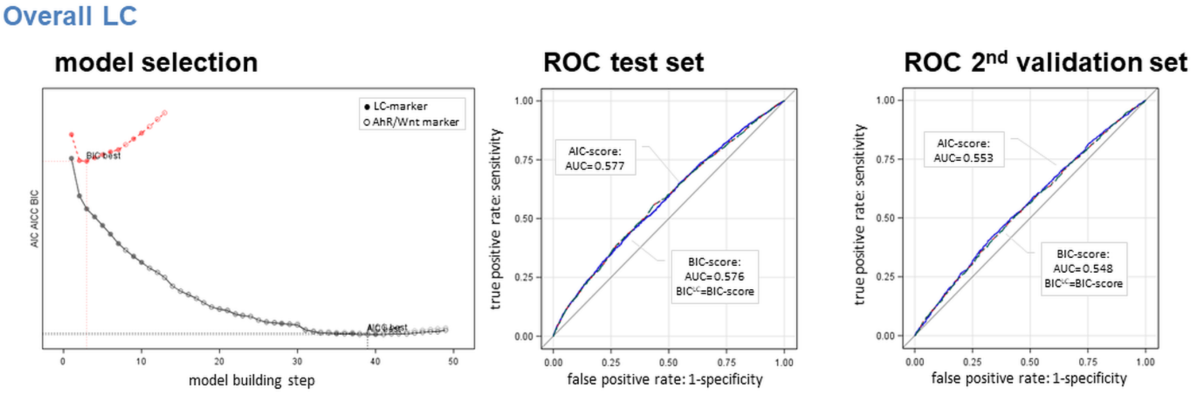

Polygenic Risk scores (PRS)

S-Table 4 Score composition and importance ratio

	score composition [LC : AhR/Wnt]				importance ratio [LC : AhR/Wnt]			
	**AIC**	**AICC**	**BIC**	**BIC^{LC}**	**AIC**	**AICC**	**BIC**	**BIC^{LC}**
Overall LC	20 : 17	like AIC	8 : --	like BIC	0.56 : 0.34	like AIC	0.36 : --	like BIC
AdenoLC	22 : 31	like AIC	6 : --	like BIC	0.91 : 1.03	like AIC	0.45 : --	like BIC
SCLC	12 : 27	like AIC	3 : --	like BIC	0.60 : 1.49	like AIC	0.20 : --	like BIC
SqCLC	15 : 14	like AIC	5 : 1	5 : -	0.58 : 0.40	like AIC	0.24 : 0.03	0.24 : --
Never smoker	15 : 23	12 : 18	-- : 2	1 : --	0.96 : 1.46	0.84 : 1.02	-- : 0.18	0.10 : --
Ever smoker	22 : 19	like AIC	7 : --	like BIC	0.68 : 0.37	like AIC	0.33 : --	like BIC
**age ≤55 yrs.**	19 : 29	18 : 19	2 : 2	2 : --	1.08 : 1.16	1.04 : 0.73	0.22 : 0.09	0.22 : --

AIC and BIC: polygenic risk scores to predict lung cancer status after marker selection according the AIC and BIC criteria, respectively; BIC^{LC}: as BIC with only *LC-markers* allowed; score composition: *number of LC-markers : number of AhR/Wnt-markers*; importance $I_g = \sum_{m \ni g} |\beta_m| \cdot MAF_m$ with MAF_m the minor allele frequency and β_m the logistic regression coefficient or marker m; importance ratio: $I_{LC-markers} : I_{AhR/Wnt-markers}$; LC: overall lung cancer, SCLC: small cell lung cancer, SqCLC: squamous cell lung cancer, AdenoLC: adenocarcinoma of the lung;

S-Table 5 Discriminability of prediction scores

		Rank corr.	AUC of ROC			p-value		Somers D		
		AIC-BIC	AIC	BIC	BIC^{LC}	AIC≥BIC	AIC≥BIC^{LC}	AIC	BIC	BIC^{LC}
**training set**	**Overall LC**	0.550	0.607	0.582	like BIC	<0.001	like BIC	0.213	0.163	like BIC
	**AdenoLC**	0.415	0.640	0.585	like BIC	<0.001	like BIC	0.281	0.171	like BIC
	**SCLC**	0.336	0.698	0.583	like BIC	<0.001	like BIC	0.395	0.167	like BIC
	**SQCLC**	0.473	0.631	0.596	0.589	<0.001	<0.001	0.262	0.193	0.178
	**Never smoker**	0.262	0.699	0.585	0.539	<0.001	<0.001	0.399	0.171	0.077
	**Ever smoker**	0.510	0.620	0.589	like BIC	<0.001	like BIC	0.240	0.178	like BIC
	**age ≤55 yrs.**	0.323	0.693	0.591	0.573	<0.001	<0.001	0.385	0.182	0.146
**validation set**	**Overall LC**	0.557	0.570	0.569	like BIC	0.41	like BIC	0.141	0.139	like BIC
	**SQCLC**	0.483	0.555	0.556	like BIC	0.54	like BIC	0.110	0.111	like BIC
	SCLC	0.335	0.535	0.535	like BIC	0.51	like BIC	0.070	0.071	like BIC
	**AdenoLC**	0.429	0.594	0.593	0.593	0.48	0.48	0.188	0.187	0.187
	**Never smoker**	0.243	0.521	0.516	0.520	0.40	0.48	0.042	0.032	0.039
	**Ever smoker**	0.523	0.574	0.571	like BIC	0.30	like BIC	0.149	0.143	like BIC
	**age ≤55 yrs.**	0.313	0.546	0.576	0.591	0.98	1.00	0.092	0.152	0.181
**test set**	**Overall LC**	0.549	0.577	0.576	like BIC	0.43	like BIC	0.154	0.153	like BIC
	**AdenoLC**	0.428	0.578	0.578	like BIC	0.52	like BIC	0.156	0.157	like BIC
	**SQCLC**	0.471	0.553	0.572	like BIC	0.99	like BIC	0.106	0.144	like BIC
	**SCLC**	0.330	0.577	0.546	0.546	**0.019**	**0.019**	**0.154**	**0.092**	**0.092**
	**Never smoker**	0.243	0.540	0.551	0.514	0.71	0.13	0.079	0.103	0.028
	**Ever smoker**	0.512	0.576	0.584	like BIC	0.93	like BIC	0.152	0.169	like BIC
	**age ≤55 yrs.**	0.324	0.600	0.575	0.593	0.039	0.31	0.200	0.150	0.186
**2nd validation set**	**Overall LC**	0.565	0.553	0.548	like BIC	0.17	like BIC	0.107	0.095	like BIC
	AdenoLC	0.412	0.561	0.570	like BIC	0.82	like BIC	0.122	0.141	like BIC
	**SCLC**	0.370	0.564	0.531	like BIC	**0.08**	like BIC	**0.129**	**0.063**	like BIC
	**SQCLC**	0.463	0.564	0.575	0.575	0.85	0.85	0.128	0.150	0.150
	**Never smoker**	0.138	0.535	0.526	0.526	0.36	0.36	0.070	0.053	0.053
	**Ever smoker**	0.527	0.564	0.562	like BIC	0.39	like BIC	0.128	0.124	like BIC
	**age ≤55 yrs.**	0.286	0.525	0.556	0.556	0.91	0.91	0.049	0.112	0.112

AIC and BIC: polygenic risk scores to predict lung cancer status after marker selection according the AIC and BIC criteria; Rank corr: Spearman's rank correlation coefficient between the AIC- and the BIC-score. AUC of ROC: area under the ROC-curve; randomly partitioning the sample (14,068 LC-cases and 12,390 controls) into a training set, validation set and test set by the ration ⅓:⅓:⅓. 2nd validation set: independent sample of size n=4,359; LC: overall lung cancer, SCLC: small cell lung cancer, SqCLC: squamous cell lung cancer, AdenoLC: adenocarcinoma of the lung

S-Figure 4 LC-risk score: model selection and ROCs for SCLC and Never smoker

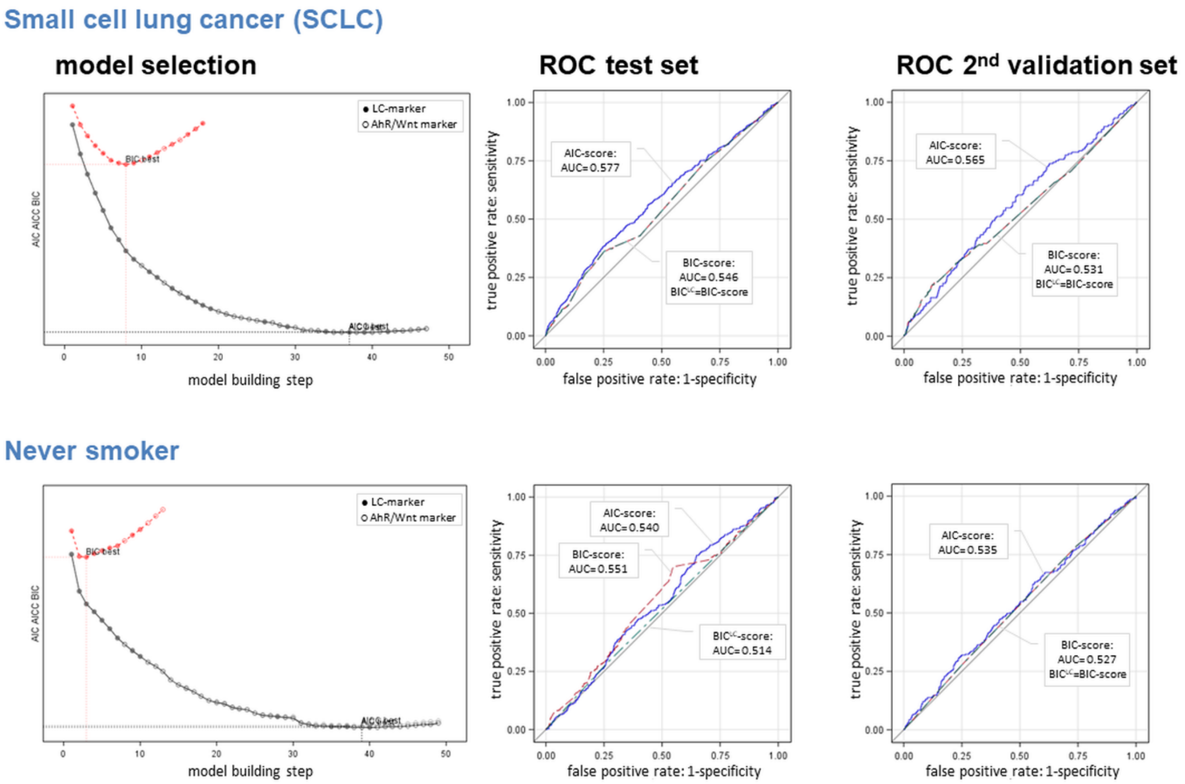

Decision trees

S-Table 6 Prediction accuracy of the decision trees

	LC-cases$^{\$}$	*con-trols$^{\$}$*	*Complex-ity &*	*Somers' D*	*95%-CI*	*TP rate*	*TN rate*
LC	2,360	1,999	[1 / 0] / 1	0.0565	[0.0272; 0.0857]	62,1%	43,6%
SCLC	164	1,999	[1 / 1] / 2	-0.0005	[-0.0015; 0.0005]	0%	99,9%
SqCLC	614	1,999	[4 / 5] / 4	0.0126	[0.0019; 0.0233]	1,8%	99,5%
AdenoLC	--	--	[0 / 0] / 0	--	--	--	--
Ever smokers	2,053	1,165	[5 / 1] / 4	0.007	[-0.0073; 0.0221]	96,1%	4,6%
Never smokers	307	834	[2 / 7] / 4	-0.002	[-0.0225; 0.0195]	2,6%	97,2%
age ≤55 yrs.	343	440	[1 / 0] / 1	0.096	[0.0270; 0.1645]	64,1%	45,5%

LC: overall lung cancer, SCLC: small cell lung cancer, SqCLC: squamous cell lung cancer, AdenoLC: adenocarcinoma of the lung; $ of the 2nd validation set; TP rate: true positive rate (sensitivity), TN rate: true negative rate (specificity); & The complexity (nodes/depth) of a decision tree is given as [number of nodes by *LC-markers* / number of nodes by *AhR/Wnt-markers*] / number of levels.

S-Table 7 Prediction accuracy in never smokers by histological subtypes

Branch		LC		SCLC		SqCLC		adenoLC		other	
**Training**	entire sample										
**I**	I (mixed)	653	61%	20	3%	58	9%	328	50%	247	38%
**II**	II (mixed)	129	12%	5	4%	9	7%	76	59%	39	34%
**III**	III (affected)	52	5%	2	4%	4	8%	30	58%	16	31%
	IV (unaffected)	235	22%	7	3%	20	9%	129	55%	79	34%
p-value=	*0.7416*	*1096*	*100%*								
**Testing**	2nd validation set										
**I**	I (mixed)	183	59%	11	6%	30	16%	96	52%	46	25%
**II**	II (mixed)	27	9%	1	4%	3	11%	16	59%	7	26%
**III**	III (affected)	8	3%	0	--	2	25%	5	63%	1	13%
	IV (unaffected)	89	29%	2	2%	12	13%	60	67%	15	17%
p-value=	*0.5437*	*307*	*100%*								

LC: overall lung cancer, SCLC: small cell lung cancer, SqCLC: squamous cell lung cancer, AdenoLC: adenocarcinoma of the lung; p-value of Fisher's Exact Test; I: MTAP<0.0385 and *TERT*<2; II: MTAP<0.0385 and *TERT*>=2; III: MTAP>=0.0385 (affected); IV: MTAP>=0.0385 (unaffected);

S-Figure 5 Decision tree for early onset LC (age ≤55 yrs.)

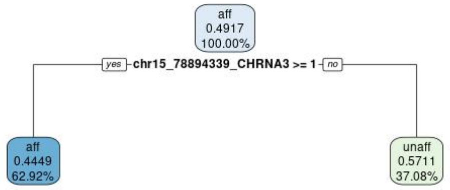

Node information: "unaff"=control (unaffected), "aff"=LC-case (affected), percentage of true decision, portion of sample rated at this node (sums to 100% across each level). Colour code refers to the decision: green=affected, blue=unaffected; split information below the node: marker, gene and threshold for minor allele count.

S-Figure 6 Decision tree for SqCLC

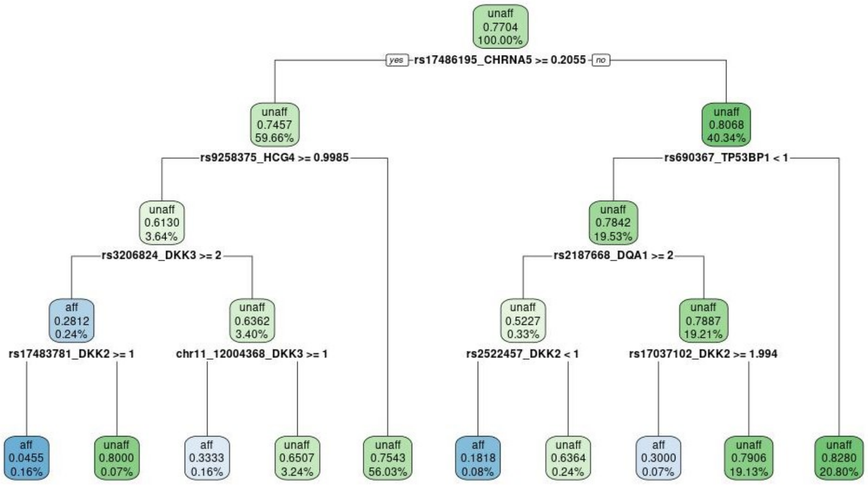

Node information: "unaff"=control (unaffected), "aff"=LC-case (affected), percentage of true decision, portion of sample rated at this node (sums to 100% across each level). Colour code refers to the decision: green=affected, blue=unaffected; split information below the node: marker, gene and threshold for minor allele count.

S-Figure 7 Decision tree for SCLC

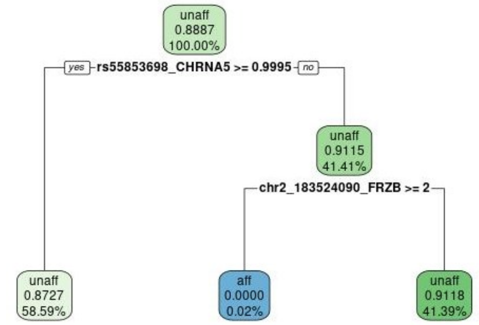

Node information: “unaff”=control (unaffected), “aff”=LC-case (affected), percentage of true decision, portion of sample rated at this node (sums to 100% across each level). Colour code refers to the decision: green=affected, blue=unaffected; split information below the node: marker, gene and threshold for minor allele count.

S-Figure 8 Decision tree for ever smoker

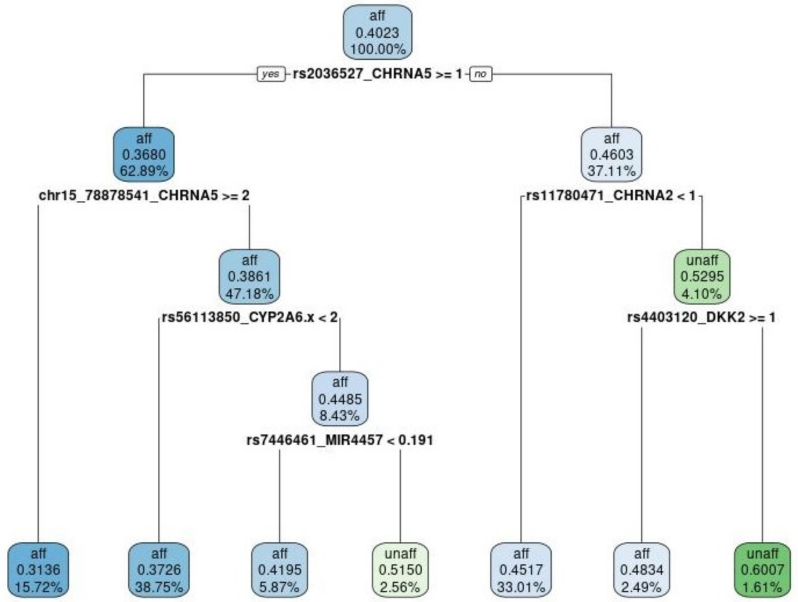

Node information: “unaff”=control (unaffected), “aff”=LC-case (affected), percentage of true decision, portion of sample rated at this node (sums to 100% across each level). Colour code refers to the decision: green=affected, blue=unaffected; split information below the node: marker, gene and threshold for minor allele count.

Expression in normal tissue

Expression profile information according to the Human Protein Atlas [4]

AHR, *Axin2*, *DKK3* can be described as 'multiplayer', with RNA expression detected in many tissue and evidence for protein existence. *DKK2* expression is uncertain, but RNA expression is enhanced in female cervix and uterine, and male and female lung. *DKK3* is strongest expressed female brain and male muscle tissue. *DKK4* expression is uncertain, but RNA expression is enhanced in the oesophagus and the gallbladder. *SFRP4* RNA expression is enhanced in the cervix, uterine, endometrium and the retina.

S-Figure 9 Expression profiles according to the Human Protein Atlas

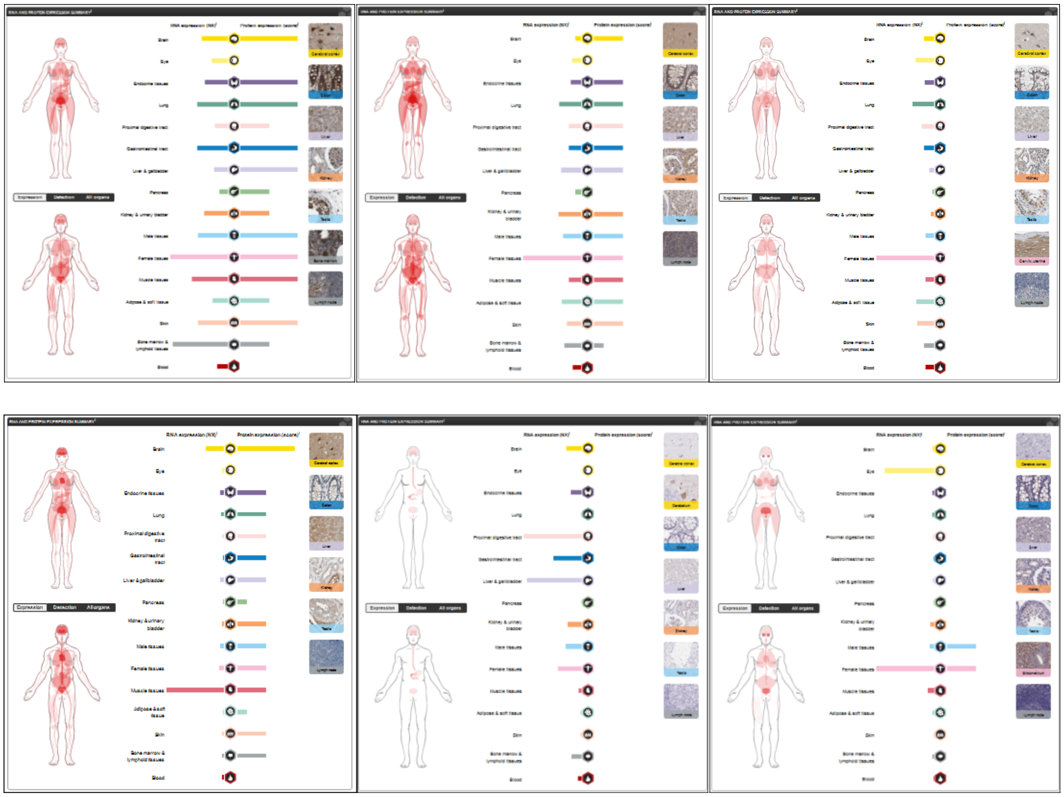

First row: *Axin2*; *AhR*; *DKK2*; second row: *DKK3*, *DKK4*, *SFRP4*; Human Protein Atlas [5]

S-Table 8 Expression in normal tissue of the lung according LungGENS

Gene	***Cell type***	***Frequency***	***Fold-change***	***DE test -log(p)***
AHR	Myolid	19.33%	2.294	6.18
	VascularEndo	14.87%	1.866	7.13
	AT1	15.61%	1.743	1.42
Axin2	VascularEndo	7.29%	1.171	2.53
DKK2	ArterialEndo	33.78%	65	34
DKK3	MatrixFB-1	14.13%	2.254	8.30
DKK4$	--			
SFRP4	Matrix Fb-2	19.96%	15.39	-13.37

The table summarizes gene expression data from Lung Gene Expression Analysis (LungGen). [6, 7] (Species: Human; Phase: Dropseq *PND1*; single gene query); if p-value<0.05 [equal to -ln(p)>1.3; Frequency: Number of expressed cells / total number of cells in cluster. DE differential expression; AT1: Pulmonary alveolar type I (AT1) cells; MatrixFB-1/2, Matrix Fibroblast type ½: subtype of Mesenchymal Cell; VascularEndo: *Vascular endothelial cells;* ArterialEndo: arterial endothelial cells; $ *DKK4* no information available

S-Table 9 **Expression in normal tissue of the lung according *The Human Protein Atlas***

Gene	*Cell type*	*Level*	*Reliability*
AHR	alveolar cells	Not detected	Enhanced
AHR	macrophages	Low	Enhanced
Axin2	alveolar cells	Medium	Approved
Axin2	macrophages	High	Approved
DKK2	alveolar cells	Not detected	Uncertain
DKK2	macrophages	Not detected	Uncertain
DKK3	alveolar cells	Medium	Approved
DKK3	macrophages	Medium	Approved
DKK4	alveolar cells	Not detected	Uncertain
DKK4	macrophages	Not detected	Uncertain
SFRP4	alveolar cells	Not detected	Enhanced
SFRP4	macrophages	Not detected	Enhanced

This table is extracted from the Human Protein Map. [4]

Level: For each of the three transcriptomics datasets (HPA, GTEx and FANTOM5), the average **Transcripts Per Million** (TPM) value of all individual samples for each human tissue or human cell type was used to estimate the gene expression level. The resulting transcript expression values, denoted Normalized eXpression (NX), were calculated for each gene in every sample.

Explanation of the specificity category

Category	Description
**Enriched**	NX level in a particular tissue/region/cell type at least four times any other tissue/region/cell type
**Group enriched**	NX levels of a group (of 2-5 tissues or 2-10 cell types or 2-5 brain regions) at least four times any other tissue/region/cell type
**Enhanced**	NX levels of a group (of 1-5 tissues or 1-10 cell types or 1-5 brain regions) at least four times the mean of other tissue/region/cell types
**Low specificity**	NX ≥ 1 in at least one tissue/region/cell type but not elevated in any tissue/region/cell type
**Not detected**	NX < 1 in all tissue/cell/region types

Reliability: The manually set reliability score indicates the level of reliability of the analysed protein expression pattern based on knowledge-based evaluation of available RNA-seq data, protein/gene characterization data and immunohistochemically data from one or several antibodies designed towards non-overlapping sequences of the same gene. The reliability score is based on the 44 normal tissues analysed and is displayed on both the Tissue Atlas and the Pathology Atlas.

The reliability score is divided into Enhanced, Supported, Approved, or Uncertain.

References

1. Bahl C, Singh N, Behera D, Sharma S (2017) Association of polymorphisms in Dickopff (DKK) gene towards modulating risk for lung cancer in north Indians. Future Oncol 13:213–232

2. Bahl C, Singh N, Behera D, Sharma S (2017) High-order gene interactions between the genetic polymorphisms in Wnt and AhR pathway in modulating lung cancer susceptibility. Personalized Medicine. https://doi.org/10.2217/pme-2017-0018

3. Machiela MJ, Chanock SJ (2015) LDlink: a web-based application for exploring population-specific haplotype structure and linking correlated alleles of possible functional variants. Bioinformatics 31:3555–3557

4. Uhlén M, Fagerberg L, Hallström BM, et al (2015) Tissue-based map of the human proteome. Science. https://doi.org/10.1126/science.1260419

5. The Human Protein Atlas. https://www.proteinatlas.org/. Accessed 5 Feb 2021

6. Du Y, Kitzmiller JA, Sridharan A, et al (2017) Lung Gene Expression Analysis (LGEA): an integrative web portal for comprehensive gene expression data analysis in lung development. Thorax 72:481–484

7. Du Y, Guo M, Whitsett JA, Xu Y (2015) “LungGENS”: a web-based tool for mapping single-cell gene expression in the developing lung. Thorax 70:1092–1094
